# Neurodevelopment Among Infants With Late-Onset Fetal Growth Restriction

**DOI:** 10.1001/jamanetworkopen.2025.17360

**Published:** 2025-06-25

**Authors:** Liqun Sun, Fu-Tsuen Lee, Natasha Milligan, Mengyuan Zhu, Joshua F. P. van Amerom, Brahmdeep S. Saini, Jessie Mei Lim, Christopher K. Macgowan, Edmond Kelly, John C. Kingdom, Mike Seed

**Affiliations:** 1 Institute of Medical Genetics and Development, Key Laboratory of Reproductive Genetics (Ministry of Education) and Women’s Hospital, Zhejiang University School of Medicine, Hangzhou, China; 2 Department of Ultrasound, Women’s Hospital, Zhejiang University School of Medicine, Hangzhou, China; 3 Division of Cardiology, Department of Pediatrics, The Hospital for Sick Children, University of Toronto, Toronto, Ontario, Canada; 4 Translational Medicine Program, SickKids Research Institute, Toronto, Ontario, Canada; 5 Department of Physiology, Temerty Faculty of Medicine, University of Toronto, Toronto, Ontario, Canada; 6 Department of Medical Biophysics, Temerty Faculty of Medicine, University of Toronto, Toronto, Ontario, Canada; 7 Department of Neonatology, Mount Sinai Hospital, University of Toronto, Toronto, Ontario, Canada; 8 Department of Obstetrics and Gynecology, Mount Sinai Hospital, University of Toronto, Toronto, Ontario, Canada; 9 Department of Diagnostic Imaging, The Hospital for Sick Children, University of Toronto, Toronto, Ontario, Canada

## Abstract

**Importance:**

Fetal growth restriction (FGR) is associated with adverse neurodevelopmental outcomes. However, the delineation of neurodevelopmental sequela in late-onset FGR has been hampered by challenges in diagnosing late-onset FGR and the confounding influence of prematurity.

**Objective:**

To characterize neurodevelopmental outcomes in full-term infants exposed to late-onset FGR and to examine the association of FGR with fetal hemodynamics, perinatal brain development, and somatic growth.

**Design, Setting, and Participants:**

In this single-center cohort study, pregnant persons with fetuses small for gestational age were enrolled between April 1, 2010, and August 31, 2016, and followed up until the infant was 36 months of age. Follow-up was completed November 2019. Data analysis was performed from June to August 2024.

**Exposures:**

Late-onset FGR diagnosed based on a composite scoring system.

**Main Outcomes and Measures:**

The primary outcomes were neurodevelopmental outcomes at 4, 8, and 12 months of age assessed by the Alberta Infant Motor Scale (AIMS) and at 18 and 36 months of age assessed by the Bayley Scales of Infant and Toddler Development, Third Edition. Secondary outcomes included fetal hemodynamics and perinatal brain development assessed by magnetic resonance imaging findings and serial somatic growth.

**Results:**

Among 97 singleton pregnancies (mean [SD] maternal age, 33.5 [3.8] years; 50 [52%] male neonates), 41 neonates (42%) were exposed to late-onset FGR. At 12-month follow-up, motor development was significantly delayed among full-term infants exposed to late-onset FGR compared with neonates appropriate for gestational age (AIMS mean difference, −4.5; 95% CI, −8.6 to −0.3). At all other time points, neurodevelopmental outcomes were similar between the groups. In models adjusted for covariates, gestational age at birth was associated with 18-month cognitive outcomes (coefficient, 4.13 [95% CI, 0.54-7.72]), while the diagnosis of late-onset FGR was not. Preterm infants exposed to FGR exhibited higher fetal combined ventricular output, higher ratio of cerebral to pulmonary blood flow, and lower oxygen saturation compared with full-term infants exposed to FGR and infants with no FGR exposure. In general, neonatal brain maturation and somatic growth by 12 months of age were similar between full-term infants exposed to FGR and those with no exposure. However, head circumference was smaller from birth until the 36-month follow-up in infants exposed to FGR.

**Conclusions:**

In this cohort study, full-term infants exposed to late-onset FGR exhibited normal neurodevelopmental outcomes by 18 and 36 months of age, and longer gestation was associated with improved outcomes. These findings suggest that early delivery is unlikely to offer neurodevelopmental benefit, and any adverse impact on neurodevelopmental outcomes of late-onset FGR among full-term infants is likely to be modest.

## Key Points

**Question:**

Is late-onset fetal growth restriction (FGR) without preterm birth associated with adverse early neurodevelopmental outcomes?

**Findings:**

In this cohort study of 97 infants, full-term infants exposed to late-onset FGR had neurodevelopmental outcomes at 18 and 36 months of age similar to those of infants with no FGR exposure. Longer gestation was associated with favorable cognitive outcomes at 18 months of age.

**Meaning:**

These findings suggest that, in the absence of absolute indications for early delivery, safely advancing gestational age in late-onset FGR may be associated with improvements in early neurodevelopmental outcomes, and any adverse impact on neurodevelopmental outcomes of full-term infants exposed to FGR is likely to be modest.

## Teaser

This cohort study characterizes early neurodevelopmental outcomes and identifies modifiable risk factors for adverse neurodevelopmental outcomes among full-term infants exposed to late-onset fetal growth restriction.

## Introduction

Fetal growth restriction (FGR) is a common pregnancy complication and remains one of the primary contributors to perinatal mortality, as well as short- and long-term morbidity.^1,2^ In particular, there is broad agreement that individuals exposed to FGR are at a notable risk for early adverse neurodevelopmental outcomes.^3,4,5^ However, the recent classification of early- and late-onset FGR has highlighted distinct clinical and pathophysiological characteristics, with late-onset FGR typically associated with a milder presentation, which may be associated with a different neurodevelopmental phenotype.^6,7^

There are currently no effective treatments for placental dysfunction, and the focus of care remains enhanced fetal surveillance and delivery based on absolute and relative indications. However, international guidelines regarding the optimal timing of delivery in late-onset FGR vary considerably,^8,9^ and there is no conclusive evidence whether Doppler evaluation of cerebroplacental redistribution is an appropriate indication for delivery, especially when compared with the potential benefits of safely advancing gestational age from a neurodevelopmental perspective.^9,10^ Moreover, it is unknown whether postnatal catch-up growth in infants affected by late-onset FGR mitigates any adverse prenatal impact on brain development.^11^ By contrast, preterm delivery is associated with a range of complications, including neurodevelopmental morbidity and issues relating to lung and gut immaturity.^12^ The burden of managing these complications falls on neonatal health care services, with significant costs associated with the care of late preterm infants delivered early for late-onset FGR.^13^ Therefore, establishing the relative importance of prenatal and postnatal influences on neurodevelopmental outcomes is of critical importance for the optimization of perinatal management in the setting of late-onset FGR. The objective of this observational cohort study was to longitudinally characterize early neurodevelopmental outcomes in full-term infants exposed to late-onset FGR and identify any modifiable risk factors for adverse neurodevelopmental outcomes in this population.

## Methods

### Study Design and Setting

This prospective observational cohort study evaluated fetal circulatory physiology and neurodevelopmental outcomes in infants exposed to FGR and unaffected controls. The primary objective was to longitudinally assess early neurodevelopmental outcomes at 4, 8, and 12 months using the Alberta Infant Motor Scale (AIMS) and at 18 and 36 months using the Bayley Scales of Infant and Toddler Development, Third Edition (BSID-III). The secondary objective was to assess the associations among fetal circulatory physiology, perinatal brain development, early somatic growth, and infant neurodevelopmental outcomes. The study was conducted at Mount Sinai Hospital and The Hospital for Sick Children, Toronto, Ontario, Canada, with the recruitment period spanning from April 1, 2010, to August 31, 2016, and last follow-up visit November 2019. This study received research ethics board approval from both institutions and adhered to the Good Clinical Practice Guidelines of the International Council of Harmonisation^14^ and the Principles of the Declaration of Helsinki.^15^ All participating pregnant women provided written informed consent. This study followed the Strengthening the Reporting of Observational Studies in Epidemiology (STROBE).

### Study Participants

Pregnant women were recruited from high- and low-risk antenatal clinics at Mount Sinai Hospital. Eligible participants for the study were 18 years or older and carrying singleton pregnancies at 32 weeks’ gestation or later with a range of estimated fetal weights but with special attention paid to the recruitment of those with an estimated fetal weight at or below the 25th percentile, with the expectation that some of these fetuses would develop late-onset FGR. Estimated fetal weight was derived using the Hadlock formula by measuring the fetal head circumference, abdominal circumference, and femur length and converted to a percentile for gestational age using an ultrasonographic fetal growth standard as recommended by the current Canadian FGR guideline.^10^ The study excluded individuals with pregnancies involving fetal structural or genetic anomalies, congenital infections, or anemia and any individuals with a contraindication for fetal magnetic resonance imaging (MRI). Enrolled pregnant participants were assessed using fetal cardiac magnetic resonance (CMR). After birth, term newborns (gestational age, ≥37 weeks) underwent neonatal brain MRI during natural sleep and were followed up at 4, 8, 12, 18, and 36 months for neurodevelopmental assessment. Both fetal CMR and neonatal brain MRI were performed on a clinical 1.5T magnetic resonance system (Avanto Fit; Siemens Medical Solutions) without contrast agents or sedation. Brain imaging was not performed on preterm neonates with late-onset FGR due to their fragile condition at the time of birth, and neurodevelopmental follow-up was discontinued in this population due to logistical reasons. Ethnicity, race, and maternal level of education were self-reported and assessed to evaluate the representativeness of the sample and potential influences on study outcomes. Ethnicity and race were categorized as Black (African, African American, or Caribbean), East Asian (Chinese, Japanese, or Korean), South Asian (Bangladeshi, Indian, or Pakastani), and White (European, Hispanic, Middle Eastern, or North African).

### Exposures

The diagnosis of FGR was established postnatally based on a previously reported composite scoring system (eMethods in Supplement 1).^16^ A score of 2 or more of the following 4 parameters was required for a diagnosis of FGR: (1) birthweight at the third percentile or less or a fetal growth curve exhibiting a 20% or greater drop in the estimated fetal weight percentile; (2) lowest cerebroplacental ratio (CPR) less than the fifth percentile after 30 weeks; (3) ponderal index less than 2.2; or (4) placental pathology diagnosis associated with FGR, principally maternal vascular malperfusion disease.^17^ Participants were then divided into 1 of 3 groups. Group 1 was defined as meeting criteria for FGR and born at a gestational age of less than 37 weeks (preterm FGR). Group 2 was defined as meeting criteria for FGR with delivery at a gestational age of 37 weeks or later (term FGR). Group 3 was born appropriate for gestational age (AGA), defined as delivery at 37 weeks or later with no evidence of FGR. Fetal Doppler parameters were obtained according to obstetric guidelines,^18^ including the umbilical artery pulsatility index (UA-PI), middle cerebral artery pulsatility index (MCA-PI), and CPR, all of which were converted to *z* scores.^19^

### Outcomes

#### Fetal CMR

Fetal CMR was performed to assess fetal circulatory physiology according to a previously published protocol^20^ including the measurement of fetal volume and fetal brain volume to produce estimated fetal body and brain weights (eMethods in Supplement 1). In brief, fetal blood flow measures included the ascending aorta, main pulmonary artery, ductus arteriosus, descending aorta, umbilical vein, and superior vena cava (SVC). Combined ventricular output was calculated as the sum of ascending aorta and main pulmonary artery plus 3% for coronary blood flow, and pulmonary blood flow consisted of the sum of right and left pulmonary artery branch flows. SVC flow was adopted as a proxy for cerebral blood flow, as there are no current methods to directly assess total fetal cerebral blood flow, and a prior study^21^ demonstrated that a predominant portion of SVC drainage was composed of cerebral blood flow. We calculated the ratio of SVC to pulmonary blood flow as a measure of the degree of centralization of the fetal circulation. Oxygen saturations were obtained in the ascending aorta, main pulmonary artery, descending aorta, umbilical vein, and SVC. Fetal blood flow, oxygen saturations, and gestational age–appropriate population means of hemoglobin concentrations were used to calculate oxygen transport parameters across the fetal circulation, including fetal oxygen delivery (Do_2_), oxygen consumption (Vo_2_), oxygen extraction, cerebral oxygen delivery (CDo_2_), cerebral oxygen consumption (CVo_2_), and cerebral oxygen extraction.

#### Neonatal Brain MRI

Neonatal brain MRI was used to assess perinatal brain growth and maturation (eMethods in Supplement 1). In brief, a 3-dimensional steady-state free precision sequence was used to obtain total brain volume, which was converted to brain weight *z* score.^22,23^ Diffusion tensor imaging was performed to measure apparent diffusion coefficient (ADC) and fractional anisotropy (FA), a measure of water diffusion and directionality that increases with brain maturation, in predetermined regions in the subcortical white matter and deep gray matter of each hemisphere. Magnetic resonance spectroscopy was performed to assess mean levels of cerebral metabolites, including N-acetylaspartate, choline, creatine, and lactate, across the basal ganglia and centrum semiovale.

#### AIMS for 4 to 12 Months of Age

Early infant neurodevelopment was assessed using the AIMS that is designed to measure early gross motor skill maturation and identify motor delays and disorders.^24^ The AIMS evaluates simple to advanced position-centric movements from prone, supine, and sitting to standing. A total of 58 movements are assessed and scores are reported based on whether each movement is observed. Possible scores are lowest at birth (mean, 4.5) and increase until 13 months of age (mean, 58.0).

#### BSID-III for 18 to 36 Months of Age

Infant and toddler neurodevelopment was assessed using the BSID-III.^25^ This standardized assessment, administered by certified professional psychometrists, is designed to measure neurodevelopmental functioning and identify potential delays and disorders. The BSID-III evaluates 3 key domains: cognitive, language, and motor development. The cognitive scale assesses how a child thinks, pays attention, reacts, and learns about their environment. The language scale assesses how a child understands and expresses language. The motor scale assesses both gross and fine motor skills. All 3 scales are reported as standardized composite scores (mean [SD], 100 [15]; range, 45-155). Somatic growth parameters of weight, height (or length), body mass index (BMI), and head circumference were collected at birth and neurodevelopmental follow-up assessments and converted to sex-specific *z* scores using the World Health Organization growth charts for Canada 2014.^26^

### Statistical Analysis

Data analysis was performed from June to August 2024. Given the hypothesis-generating nature of this study, we did not adjust for multiplicity. Results were expressed in percentages, means (SDs), medians (IQRs), or mean differences (95% CIs) between the FGR and AGA groups. Comparisons between exposure and outcome variables were assessed using the Fisher exact test for proportions and 2-sample and multiple-group comparison tests. Associations among fetal circulatory measures, somatic and brain growth, and neurodevelopmental outcomes were assessed by correlation analysis. Mixed-effect modeling with restricted maximum likelihood estimation was performed to assess longitudinal weight, length, BMI, and head circumference *z* score trajectories from birth to 36 months of age in patients who had 3 or more anthropometric measurements recorded. Multivariable linear regression was performed to examine the associations of late-onset FGR, gestational age, and biological sex with neurodevelopmental outcomes at 4, 8, 12, 18, and 36 months of age. The regression coefficient and adjusted mean differences were reported with 95% CIs for all models. A 2-sided *P* < .05 was considered statistically significant. Statistical analyses were performed using GraphPad Prism, version 10.3.1 (GraphPad Software), and R, version 4.3.2 (R Foundation for Statistical Computing).

## Results

### Study Population

**Figure 1.**
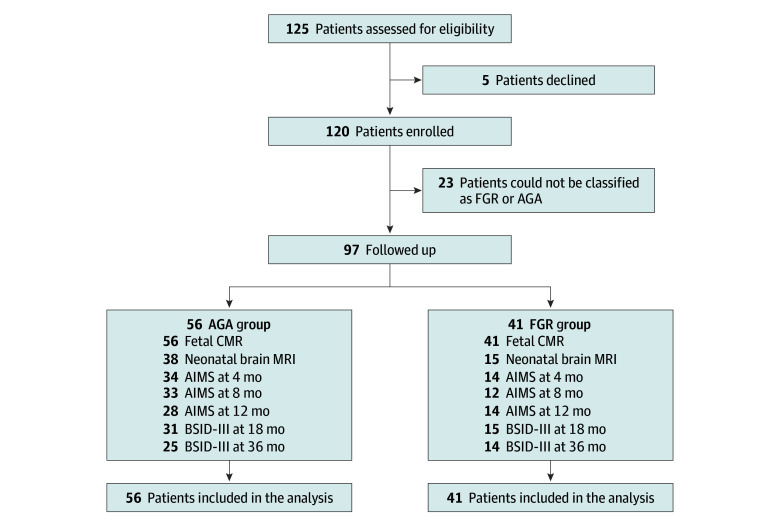
Study Flow Diagram Among participants born appropriate for gestational age (AGA), 56 were invited for follow-up. Loss to follow-up was due to participant decline. Among participants exposed to fetal growth restriction (FGR), those with term FGR were invited for neurodevelopmental follow-up. Of the 25 participants in the term FGR group, loss to follow-up was due to participant decline. AIMS indicates Alberta Infant Motor Scale; BSID-III, Bayley Scales of Infant and Toddler Development, Third Edition.

A total of 97 singleton pregnancies were studied (mean [SD] maternal age, 33.5 [3.8] years), including 47 (48%) male and 50 (52%) female neonates. Maternal race included 2 (2%) Black, 19 (20%) East Asian, 5 (5%) South Asian, 59 (61%) White, and 12 (12%) unreported. Forty-one pregnancies met the diagnostic criteria for FGR, with 16 classified as preterm FGR and 25 as term FGR. Fifty-six pregnancies were considered AGA, with all infants born at term. A description of the cohort characteristics is presented in Table 1, and the STROBE flowchart of the study is presented in Figure 1.

**Table 1.** Demographic and Medical Characteristics

Characteristic	Neonatal group			*P* value
	Preterm FGR (n = 16)	Term FGR (n = 25)	AGA (n = 56)	
Maternal				
Age at conception, mean (SD), y	33.6 (4.0)	33.8 (4.8)	33.5 (3.4)	.95
Ethnicity and race, No. (%)				
Black (African, African American, or Caribbean)	0	2 (8)	0	.18
East Asian (Chinese, Korea, or Japanese)	2 (13)	5 (20)	12 (21)	
South Asian (Bangladeshi, Indian, or Pakistani)	3 (19)	1 (4)	1 (2)	
White (European, Hispanic, Middle Eastern, or North African)	9 (56)	14 (56)	36 (64)	
Unreported	2 (13)	3 (12)	7 (13)	
Highest educational level, No. (%)				
Elementary school	0	0	0	.04
Some high school	1 (6)	0	0	
High school diploma	3 (19)	1 (4)	1 (2)	
Some university or college	0	1 (4)	0	
University or college degree	10 (63)	12 (48)	34 (61)	
Graduate or professional	0	6 (24)	8 (14)	
Unreported	2 (13)	5 (20)	13 (23)	
Fetal				
Sex, No. (%)				
Female	6 (38)	9 (36)	35 (63)	.04
Male	10 (63)	16 (64)	21 (38)	
Estimated fetal weight *z* score, mean (SD)	−2.0 (0.6)	−1.4 (0.8)	0.1 (0.8)	<.001
Estimated fetal brain weight *z* score, mean (SD)	−1.2 (0.5)	−0.5 (0.9)	−0.2 (1.0)	<.001
Postnatal				
Mode of delivery, No. (%)				
Spontaneous vaginal delivery	13 (81)	12 (48)	35 (63)	.02
Cesarean section	2 (13)	13 (52)	14 (25)	
Missing data	1 (6)	0	7 (13)	
Birthweight *z* score, mean (SD)	−4.7 (1.1)	−1.9 (0.9)	0.1 (0.9)	<.001
Length *z* score, mean (SD)	−5.1 (2.0)	−1.1 (1.7)	1.3 (1.2)	<.001
Head circumference *z* score, mean (SD)	−4.1 (1.9)	−1.2 (0.9)	0.2 (1.2)	<.001
Head circumference to birthweight ratio, mean (SD)	21.3 (4.7)	13.2 (1.6)	10.5 (1.1)	<.001
Ponderal index, mean (SD)	2.3 (0.3)	2.4 (0.3)	2.5 (0.3)	.02
Placenta weight percentile, median (IQR)	1 (1-5)	20 (2-37)	60 (35-90)	<.001
Abnormal placental histology, No. (%)				
Yes	14 (88)	17 (68)	7 (13)	<.001
No	1 (6)	4 (16)	20 (36)	
Missing data	1 (6)	4 (16)	29 (52)	
Apgar at 1 min, mean (SD)	7.1 (2.6)	8.4 (1.3)	8.6 (1.0)	.001
Apgar at 5 min, mean (SD)	8.2 (2.3)	9.0 (0.2)	9.0 (0)	.01
Cord pH, mean (SD)	7.3 (0.1)	7.3 (0.1)	7.3 (0.2)	.10
Cord base, mean (SD)	−5.6 (3.5)	−5.9 (2.8)	−5.0 (2.9)	.46
Cord Po_2_, mean (SD), mm Hg	22.5 (11.1)	24.1 (6.9)	26.6 (8.7)	.25
Cord Pco_2_, mean (SD), mm Hg	54.5 (15.0)	48.3 (12.0)	44.7 (6.0)	.007
NICU admission, No. (%)				
Yes	15 (94)	4 (16)	5 (9)	<.001
No	0	21 (84)	44 (79)	
Missing data	1 (6)	0	7 (13)	
Follow-up, mean (SD)				
Gestational age at fetal CMR, wk	33.6 (1.6)	36.9 (1.7)	36.4 (1.3)	<.001
Gestational age at birth, wk	34.4 (1.8)	38.9 (1.2)	39.4 (1.1)	<.001
Postmenstrual age at neonatal brain MRI, wk	NA	40.2 (1.7)	40.8 (2.3)	.35
Age, mean (SD), mo				
At 4-mo AIMS	NA	4.2 (0.3)	4.2 (0.6)	.97
At 8-mo AIMS	NA	8.6 (0.8)	8.4 (0.6)	.41
At 12-mo AIMS	NA	12.4 (0.6)	12.5 (0.8)	.76
At 18-mo BSID-III	NA	18.8 (1.8)	19.4 (1.7)	.24
At 36-mo BSID-III	NA	37.2 (1.0)	37.0 (0.8)	.69

Abbreviations: AGA, appropriate for gestational age; AIMS, Alberta Infant Motor Scale; BSID-III, Bayley Scales of Infant and Toddler Development, Third Edition; CMR, cardiac magnetic resonance; FGR, fetal growth restriction; MRI, magnetic resonance imaging; NA, not applicable; NICU, neonatal intensive care unit.

### Fetal Circulatory Physiology

On fetal CMR, unindexed Do_2_ was significantly reduced in neonates exposed to preterm (mean difference, −44.6 [95% CI, −54.7 to −34.5] mL/min) and term (mean difference, −12.2 [95% CI, −20.2 to −4.1] mL/min) FGR compared with the AGA cohort, with larger reductions in the preterm FGR cohort. Indexed Do_2_ and Vo_2_ were significantly reduced in the preterm FGR cohort compared with the term FGR (mean difference, −8.4 [95% CI, −12.0 to −4.8] and −1.8 [95% CI, −3.2 to −3.0] mL/min/kg, respectively) and AGA (mean difference, −9.6 [95% CI, −12.7 to −6.3] and −2.4 [95% CI, −3.6 to −1.0] mL/min/kg, respectively) cohorts. Compared with the term FGR and AGA cohorts, the preterm FGR cohort had significantly reduced unindexed CDo_2_ (mean difference, −18.3 [95% CI, −29.5 to −7.1] and −24.4 [95% CI, −34.0 to −14.3] mL/min, respectively) and unindexed CVo_2_ (mean difference, −3.1 [95% CI, −7.7 to 1.4] and −5.3 [95% CI, −9.4 to −1.1] mL/min, respectively). However, CDo_2_ and CVo_2_ indexed to fetal weight or fetal brain weight were similar among all 3 groups. The ratio of CDo_2_ to Do_2_ was significantly higher in the preterm FGR vs AGA cohorts (mean difference, 4.2 [95% CI, 2.1-6.1]), and similar between the term FGR and AGA cohorts (mean difference , 1.2 [95% CI, −0.2 to 2.6]). Fetal oxygen saturation in all the major fetal vessels was lowest in the preterm FGR cohort, followed by term FGR and AGA cohorts. On fetal ultrasonographic imaging, compared with the AGA cohort, the lowest CPR and CPR *z* score were significantly lower, and the highest UA-PI and UA-PI *z* score were significantly higher in the FGR cohort, whereas the lowest MCA-PI was significantly lower in the preterm FGR cohort and the lowest MCA-PI *z* score was significantly lower in the FGR cohort. Comparisons of fetal circulatory physiology are presented in Table 2 and eTable 1 in Supplement 1.

**Table 2.** Hemodynamic Parameters Between Fetuses With and Without FGR

Parameter^a^	Neonatal cohort, mean (SD)				Preterm FGR vs term FGR		Preterm FGR vs AGA		Term FGR vs AGA	
	Preterm FGR	Term FGR	AGA	*P* value	Mean difference (95% CI)	*P* value	Mean difference (95% CI)	*P* value	Mean difference (95% CI)	*P* value
Combined ventricular output, mL/min/kg	554 (116)	466 (84)	458 (54)	<.001	88 (39 to 137)	<.001	96 (52 to 139)	<.001	8 (−29 to 44)	.68
Main pulmonary artery flow, mL/min/kg	317 (76)	258 (58)	246 (47)	<.001	59 (22 to 94)	.001	71 (38 to 102)	<.001	12 (−15 to 38)	.38
Ascending aortic flow, mL/min/kg	210 (72)	194 (52)	201 (38)	.60	16 (−15 to 47)	.31	9 (−19 to 37)	.53	−7 (−30 to 16)	.55
SVC flow, mL/min/kg	238 (53)	148 (46)	131 (36)	<.001	90 (63 to 116)	<.001	107 (83 to 130)	<.001	17 (−3 to 37)	.10
Descending aortic flow, mL/min/kg	252 (68)	241 (48)	248 (35)	.73	11 (−17 to 39)	.44	5 (−20 to 30)	.71	−6 (−28 to 15)	.55
Pulmonary blood flow, mL/min/kg	58 (39)	63 (35)	72 (36)	.38	−5 (−32 to 22)	.71	−14 (−38 to 10)	.25	−9 (−27 to 8)	.31
Ductus arteriosus flow, mL/min/kg	260 (73)	188 (45)	177 (36)	<.001	72 (41 to 102)	<.001	83 (55 to 110)	<.001	11 (−11 to 34)	.32
Foramen ovale flow, mL/min/kg	187 (68)	144 (55)	142 (48)	.04	44 (4 to 83)	.03	45 (9 to 81)	.01	2 (−24 to 28)	.90
Umbilical vein flow, mL/min/kg	97 (27)	125 (31)	126 (27)	.002	−28 (−45 to −9)	.003	−28 (−44 to −12)	<.001	−1 (−14 to 12)	.90
Main pulmonary artery oxygen saturation, %	30 (4)	47 (8)	51 (7)	<.001	−17 (−24 to −10)	<.001	−22 (−27 to −15)	<.001	−4 (−8 to 0)	.04
Ascending aortic oxygen saturation, %	36 (8)	54 (9)	59 (8)	<.001	−18 (−24 to −11)	<.001	−24 (−29 to −17)	<.001	−5 (−10 to 0)	.03
SVC oxygen saturation, %	29 (6)	42 (8)	45 (7)	<.001	−13 (−19 to −6)	<.001	−16 (−22 to −9)	<.001	−3 (−7 to 1)	.15
Descending aortic oxygen saturation, %	34 (10)	50 (8)	52 (7)	<.001	−15 (−21 to −9)	<.001	−18 (−22 to −12)	<.001	−2 (−6 to 2)	.31
Umbilical vein oxygen saturation, %	61 (9)	75 (8)	79 (8)	<.001	−14 (−20 to −7)	<.001	−18 (−23 to −12)	<.001	−4 (−8 to 0)	.06
Cerebral pulmonary blood flow ratio	6.6 (4.8)	3.6 (3.8)	2.4 (1.8)	<.001	3.0 (0.7 to 5.1)	.009	4.2 (2.1 to 6.1)	<.001	1.2 (−0.2 to 2.6)	.10
Unindexed Do_2_, mL/min	13.5 (2.8)	46.0 (12.2)	58.2 (16.6)	<.001	−32.5 (−43.8 to −21.0)	<.001	−44.6 (−54.7 to −34.5)	<.001	−12.2 (−20.2 to −4.1)	.003
Unindexed Vo_2_, mL/min	5.7 (1.5)	15.4 (5.0)	20.0 (7.3)	<.001	−9.6 (−14.5 to −4.6)	<.001	−14.2 (−18.6 to −9.8)	<.001	−4.6 (−8.1 to −1.1)	.01
Do_2_, mL/min/kg	10.6 (2.3)	19.1 (5.0)	20.2 (4.7)	<.001	−8.4 (−12.0 to −4.8)	<.001	−9.6 (−12.7 to −6.3)	<.001	−1.1 (−3.6 to 1.3)	.36
Vo_2_, mL/min/kg	4.5 (1.2)	6.3 (1.8)	6.9 (2.0)	.003	−1.8 (−3.2 to −0.3)	.02	−2.4 (−3.6 to −1.0)	<.001	−0.6 (−1.5 to 0.4)	.29
Fetal oxygen extraction ratio	43.7 (11.6)	33.6 (8.0)	34.6 (7.1)	.004	10.1 (3.7 to 16.3)	.002	9.2 (3.5 to 14.7)	.002	−0.9 (−5.3 to 3.5)	.68
Unindexed CDo_2_, mL/min	21.6 (6.4)	40.0 (10.1)	45.8 (16.4)	<.001	−18.3 (−29.5 to −7.1)	.002	−24.2 (−34.0 to −14.3)	<.001	−5.8 (−13.8 to 2.1)	.15
Unindexed CVo_2_, mL/min	6.0 (2.6)	9.1 (4.4)	11.3 (5.6)	.03	−3.1 (−7.7 to 1.4)	.18	−5.3 (−9.4 to −1.1)	.01	−2.2 (−5.1 to 0.7)	.14
CDo_2_, mL/min/kg	16.9 (4.6)	16.9 (5.4)	15.7 (4.8)	.60	0 (−3.8 to 3.9)	.99	1.2 (−2.2 to 4.6)	.48	1.2 (−1.5 to 3.9)	.39
CVo_2_, mL/min/kg	4.8 (2.0)	3.8 (1.9)	3.8 (1.5)	.37	1.0 (−0.5 to 2.4)	.20	0.9 (−0.4 to 2.2)	.17	0 (−0.9 to 0.9)	.94
CDo_2_, mL/min/100 g	11.1 (3.6)	13.9 (3.8)	15 (4.7)	.04	−2.8 (−6.2 to 0.6)	.11	−3.9 (−6.9 to −0.8)	.01	−1.1 (−3.5 to 1.4)	.30
CVo_2_, mL/min/100 g	3.2 (1.5)	3.2 (1.5)	3.7 (1.5)	.43	0 (−1.3 to 1.3)	.97	−0.5 (−1.7 to 0.7)	.43	−0.5 (−1.3 to 0.3)	.24
Cerebral oxygen extraction ratio	26.6 (9.0)	22.6 (7.9)	24.9 (7.8)	.46	4.0 (−3.0 to 11.1)	.26	1.7 (−4.7 to 8.1)	.59	−2.3 (−6.8 to 2.2)	.31
CDo_2_ to Do_2_ ratio	1.6 (0.4)	0.9 (0.3)	0.8 (0.3)	<.001	0.7 (0.4 to 0.9)	<.001	0.8 (0.5 to 1.0)	<.001	0.1 (−0.1 to 0.2)	.57
Lowest CPR	0.9 (0.3)	1.4 (0.4)	1.7 (0.4)	<.001	−0.5 (−0.7 to −0.3)	<.001	−0.9 (−1.1 to −0.6)	<.001	−0.3 (−0.5 to −0.1)	.001
Lowest CPR *z* score	−4.4 (1.7)	−1.5 (1.1)	−0.5 (1.0)	<.001	−3.0 (−3.7 to −2.1)	<.001	−4 (−4.6 to −3.2)	<.001	−1 (−1.6 to −0.3)	.003
Highest UA-PI	1.6 (0.3)	1.2 (0.2)	1.1 (0.2)	<.001	0.3 (0.1 to 0.4)	<.001	0.5 (0.3 to 0.6)	<.001	0.1 (0.0 to 0.2)	.02
Highest UA-PI *z* score	3.0 (1.3)	1.9 (1.0)	1.0 (1.1)	<.001	1.1 (0.4 to 1.8)	.002	2 (1.4 to 2.6)	<.001	0.9 (0.3 to 1.4)	.001
Lowest MCA	1.2 (0.4)	1.5 (0.3)	1.7 (0.5)	.002	−0.2 (−0.5 to 0.02)	.08	−0.5 (−0.6 to −0.2)	<.001	−0.2 (−0.4 to 0.0)	.06
Lowest MCA *z* score	−2.2 (2.0)	−0.6 (1.5)	−0.1 (1.8)	<.001	−1.6 (−2.7 to −0.4)	.005	−2.1 (−3.1 to −1.0)	<.001	−0.5 (−1.4 to 0.4)	.29

Abbreviations: AGA, appropriate for gestational age; CDo_2_, fetal cerebral oxygen delivery; CPR, cerebroplacental ratio; CVo_2_, fetal cerebral oxygen consumption; Do_2_, fetal oxygen delivery; FGR, fetal growth restriction; MCA, middle cerebral artery; PI, pulsatility index; SVC, superior vena cava; UA, umbilical artery; Vo_2_, oxygen consumption.

^a^ Blood flow and oxygen transport parameters were unindexed or indexed to fetal weight, and cerebral oxygen transport parameters were unindexed or indexed to fetal weight or fetal brain weight (per 100 g).

### Perinatal Brain Growth and Maturation

Fetal brain weight mean (SD) *z* scores were significantly lower in the preterm FGR cohort (−1.2 [0.5]) compared with the term FGR (–0.5 [0.9]) and AGA (−0.2 [1.0]) cohorts (Table 1). Neonatal brain imaging revealed overall brain growth and maturation were similar in the term FGR and AGA cohorts. For example, total brain weight *z* scores were similar in both groups (mean difference −0.45; 95% CI, −0.96 to 0.05). The ADC and FA of specific white and deep gray matter regions of the neonatal brain, as well as the means of grouped regions of the brain, were generally similar between the term FGR and AGA cohorts. Only FA of the thalamus was significantly higher in AGA neonates compared with term FGR neonates (mean difference, 0.03 [95% CI, 0.00-0.04]). Cerebral metabolite measures were similar in term FGR and AGA groups (eTable 2 in Supplement 1).

### Anthropometric Growth Trajectories

**Figure 2.**
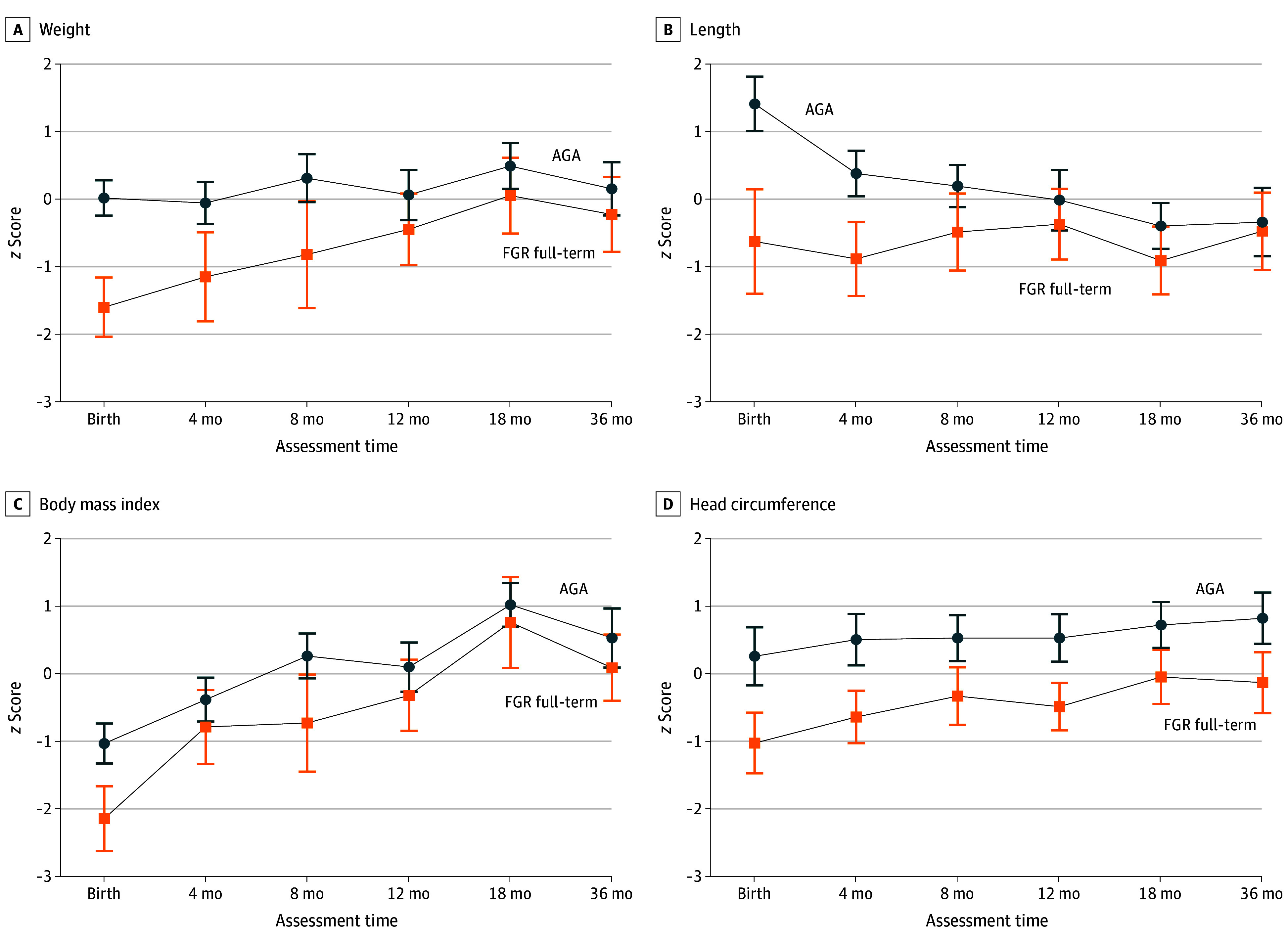
Somatic Growth Trajectories of Full-Term Infants With and Without Late-Onset Fetal Growth Restriction (FGR) Each *z* score at each time point is represented as mean (95% CI).

Weight (−1.62 [95% CI, −2.31 to −0.93]), height (−2.04 [95% CI, −3.22 to −0.85]), BMI (−1.11 [95% CI, −1.87 to −0.35]), and head circumference (−1.29 [95% CI, −2.11 to −0.46]) *z* scores at birth were significantly lower in term FGR compared with AGA cohorts. Follow-up at 4, 8, 12, 18, and 36 months revealed weight and height *z* scores converged and were similar from 12 months of age, and BMI *z* scores converged and were similar starting at 8 months of age. However, head circumference *z* scores were significantly lower from birth until follow-up at 36 months of age in the term FGR cohort compared with the AGA cohort (−0.95 [95% CI, −1.74 to −0.17]) (Figure 2 and eTable 3 in Supplement 1).

### Neurodevelopmental Outcomes

Early neurodevelopmental testing using the AIMS assessment revealed no significant differences in motor developmental milestone achievement at 4 and 8 months of age between the term FGR and AGA cohorts. Motor development at 12 months of age was significantly delayed in the term FGR compared with the AGA cohorts (mean difference, −4.5 [95% CI, −8.6 to −0.3]). Infant neurodevelopmental testing using the BSID-III revealed no significant differences in cognitive, language, and motor development at 18 and 36 months of age between the term FGR and AGA cohorts, although there was some loss to follow-up resulting in a smaller sample size (eTable 4 in Supplement 1).

### Exposure and Outcome Associations

Associations among fetal circulatory physiology, perinatal brain growth, somatic growth, and neurodevelopmental outcomes are presented in eFigures 1 to 6 in Supplement 1. There were positive associations between indexed and unindexed Do_2_ and Vo_2_ and between fetal weight and fetal weight *z* score. There were inverse associations between SVC flow and UA-PI and fetal brain weight and positive associations between CPR, CDo_2_ to Do_2_ ratio, Do_2_, Vo_2_, unindexed CDo_2_, and unindexed CVo_2_ and fetal brain weight. There were no associations between MCA-PI or fetal cerebral to pulmonary blood flow ratio and fetal brain weight. There were positive associations between umbilical vein arterial oxygen saturation (Sao_2_), ascending aorta Sao_2_, unindexed CDo_2_, unindexed CVo_2_, and cerebral oxygen delivery to fetal oxygen delivery ratio with neonatal brain weight *z* score. There were no associations between fetal Doppler indexes and neonatal brain weight *z* score. Few associations were found between fetal circulatory parameters and ADC, FA, or cerebral metabolites in the neonatal brain. There were positive associations between neonatal brain N-acetylaspartate to choline ratio and AIMS outcome at 4, 8, and 12 months of age and Bayley cognitive composite score at 18 months of age. There was an association between UA-PI *z* score with Bayley motor composite at 18 months. However, CPR, CPR *z* score, MCA, and MCA-PI were not associated with neurodevelopmental outcomes.

In models adjusted for covariates, late-onset FGR was generally not associated with any AIMS or BSID-III outcomes, except for 12-month AIMS (−4.34 [95% CI, −8.44 to −0.23]) (Table 3). However, gestational age at birth was associated with 8-month AIMS (4.50 [95% CI, 2.10-6.89]) and 18-month BSD-III cognitive composite scores (4.13 [95% CI, 0.54-7.72]). Sex was not associated with any AIMS or BSID-III outcomes. To address the possibility that the reduced sample size available at follow-up was affecting the results, a sensitivity analysis was performed comparing characteristics associated with loss to follow-up (eTables 5-11 in Supplement 1). After accounting for missing data, maternal educational level was associated with neurodevelopmental follow-up, with higher education levels associated with improved attendance.

**Table 3.** Multivariable Models Examining Associations of Full-Term Infants With FGR and Longitudinal Neurodevelopmental Outcomes

Model	Coefficient (95% CI)	*P* value
**4-mo AIMS**		
FGR	−0.89 (−3.51 to 1.73)	.49
GA	0.85 (−0.23 to 1.92)	.12
Female	0.42 (−1.94 to 2.78)	.72
**8-mo AIMS**		
FGR	−1.43 (−7.14 to 4.27)	.61
GA	4.50 (2.10 to 6.89)	<.001
Female	−1.50 (−6.50 to 3.50)	.54
**12-mo AIMS**		
FGR	−4.34 (−8.44 to −0.23)	.03
GA	1.69 (−0.17 to 3.55)	.07
Female	−0.12 (−4.01 to 3.76)	.94
**18-mo BSID-III**		
Cognitive composite		
FGR	−4.77 (−12.96 to 3.41)	.24
GA	4.13 (0.54 to 7.72)	.02
Female	2.59 (−5.20 to 10.39)	.50
Language composite		
FGR	−8.60 (−21.80 to 4.59)	.19
GA	3.02 (−2.76 to 8.79)	.29
Female	10.49 (−2.07 to 23.06)	.09
Motor composite		
FGR	−2.29 (−9.60 to 5.03)	.53
GA	3.13 (−0.07 to 6.33)	.05
Female	0.77 (−6.19 to 7.73)	.82
**36-mo BSID-III**		
Cognitive composite		
FGR	−1.08 (−7.81 to 5.65)	.74
GA	−1.92 (−4.99 to 1.16)	.21
Female	6.01 (−0.69 to 12.72)	.07
Language		
FGR	−4.84 (−13.30 to 3.63)	.25
GA	1.60 (−2.32 to 5.52)	.41
Female	7.73 (−0.85 to 16.32)	.07

Abbreviations: AIMS, Alberta Infant Motor Scale; BSID-III, Bayley Scales of Infant and Toddler Development, Third Edition; FGR, fetal growth restriction; GA, gestational age.

## Discussion

In this prospective observational cohort study, neurodevelopmental outcomes of full-term infants exposed to late-onset FGR were reassuringly similar to population means and comparable to those of full-term infants born AGA at 18 and 36 months of age. Notably, multivariable modeling identified that later gestation at birth was associated with improved infant neurodevelopmental outcomes. Our findings suggest that serial ultrasonographic-based fetal health assessments, designed to safely delay delivery for suspected late-onset FGR, may confer improved outcomes and seem unlikely to impose any increase in the risk of adverse neurodevelopmental outcomes.

During the past decade, there have been significant advancements in the diagnostic accuracy of FGR that have led to increasingly distinct perinatal management strategies for early- and late-onset FGR.^1,6,9,10,27^ However, any differences in the neurodevelopmental phenotype between early- and late-onset FGR remain poorly defined.^3,4,5^ Furthermore, lack of diagnostic clarity between FGR and healthy fetuses small for gestational age has the potential to complicate the interpretation of neurodevelopmental findings. A false-positive diagnosis of FGR has the potential to inflict harm on a healthy fetus small for gestational age via unnecessary iatrogenic preterm delivery^28^ and can therefore confound clinician attempts to minimize perinatal morbidity attributable to suspected FGR.^5,6^

The recommended indications and optimal timing of delivery in the setting of late-onset FGR are currently based on relatively limited evidence compared with early-onset FGR.^9,29,30^ Some institutions and guidelines recommend the use of MCA Doppler imaging or CPR to time delivery in late-onset FGR.^8,9,10,27^ However, the results of studies reporting on associations between MCA Doppler imaging and CPR with perinatal and neurodevelopmental outcomes have been inconsistent.^9,27,31^ In our study, the decision to deliver fetuses exhibiting evidence of late-onset FGR was largely based on the development of maternal hypertension, which in turn is associated with underlying placental dysfunction. In those fetuses with FGR who were born at term, we found no associations between MCA Doppler findings or CPR with neurodevelopmental outcomes. Moreover, MCA Doppler findings and CPR were seldom associated with any perinatal brain macrostructural or microstructural developmental findings. Indeed, most perinatal brain developmental measures and neurodevelopmental outcomes were similar between full-term infants affected by late-onset FGR and infants born AGA. Our findings would therefore be in keeping with the concept that increases in cerebral blood flow in the setting of reduced oxygen saturation protects cerebral oxygenation and brain development,^32^ although the inverse association we observed between SVC flow and fetal brain size suggests this protective mechanism may be limited. Moreover, the impact of modest late-onset FGR on body size is mitigated by postnatal catch-up growth within the first year of life. By contrast, despite some evidence of cerebral catch-up growth, fetuses with normal growth continued to exhibit larger head sizes even at 36 months. Despite smaller head size in infants with FGR, neurodevelopmental outcomes were similar to those in infants born AGA by 18 and 36 months of age, suggesting intracranial brain growth and maturation may be more suitable determinants of infant neurodevelopmental outcomes than head size.^33^

The primary goal of ultrasonographic-based fetal monitoring is to prevent fetal demise and to ensure delivery of the fetus in the best possible condition for both immediate postnatal adaptation and long-term health outcomes.^30^ This includes an individualized approach based on the condition of the fetus and the needs of the family.^29^ Our findings suggest that, in addition to the current absolute indications, an estimated fetal weight less than the third percentile to prevent perinatal death,^34^ and potential utility of CPR Doppler imaging (<5th percentile) for delivery to prevent severe neonatal morbidity based on recent evidence from the RATIO37 trial,^35^ safely advancing gestational age to prolong in utero brain development may be beneficial for infant neurodevelopmental outcomes in this respect. Furthermore, postnatal environmental influences such as reducing barriers to early developmental support may improve neurodevelopmental outcomes.^36,37^ Our findings call for future large-scale trials with safety-net criteria to determine whether delaying delivery has neurodevelopmental benefit in the setting of late-onset FGR. It is noteworthy that we observed differences in the fetal circulation between preterm and full-term infants exposed to FGR. Preterm infants with FGR demonstrated more profound circulatory centralization of blood flow in favor of the brain and heart, reflected by an increase in combined ventricular output and redistribution of cerebral to pulmonary blood flow. This compensatory mechanism was likely a response to the hypoxemia observed in all the major fetal vessels aimed at preserving vital organ function.^38^ This may explain the markedly increased ratio of head circumference to birthweight observed in infants born preterm with late-onset FGR, suggesting asymmetric growth, compared with full-term infants, who displayed more symmetric growth similar to infants born AGA. Therefore, fetal CMR could have an adjunct role in delivery planning for late-onset FGR.

### Limitations

This study has limitations. One was that multivariable modeling was constrained to only a few variables, as sample sizes were limited due to loss to follow-up. With the predominant enrollment and follow-up of mothers with a background of higher educational levels, infants of mothers with less educational attainment may have not been adequately represented, limiting the generalizability of our findings. Maternal educational level was not adjusted for in the model due to the percentage of missing data and potential overfitting in the models. Despite these limitations, we were able to show gestational age at birth appears to be a more important consideration for early neurodevelopmental outcomes than an isolated diagnosis of uncomplicated late-onset FGR. It is possible that with a larger sample size, a modest adverse association of late-onset FGR with neurodevelopmental outcomes among full-term infants may have been detected. Another limitation was that brain imaging was not performed on preterm neonates with late-onset FGR due to their fragile condition at the time of birth, and neurodevelopmental follow-up was discontinued in this population due to logistical reasons.

## Conclusions

In this cohort study, full-term infants exposed to late-onset FGR exhibited evidence of brain-sparing physiology and catch-up growth that was associated with comparable neurodevelopmental outcomes to infants born AGA at 18 and 36 months. Longer gestation was independently associated with improved neurodevelopmental outcomes, suggesting that in the absence of other indications to deliver, conservative management is not expected to incur any neurodevelopmental penalty. Any adverse impact of late-onset FGR on neurodevelopmental outcomes in infants born at term is likely to be modest.

## Notes

Supplement 1. **eMethods.** Additional Details **eTable 1.** Additional Fetal Hemodynamic Parameters **eTable 2.** Neonatal Brain MRI Outcomes **eTable 3.** Comparison of Somatic Growth Trajectories Between Those of Term Infants With and Without FGR **eTable 4.** Neurodevelopmental Outcomes **eTable 5.** Sensitivity Analysis of 4-Month Follow-Up Attendance **eTable 6.** Sensitivity Analysis of 8-Month Follow-Up Attendance **eTable 7.** Sensitivity Analysis of 12-Month Follow-Up Attendance **eTable 8.** Sensitivity Analysis of 18-Month Follow-Up Attendance **eTable 9.** Sensitivity Analysis of 36-Month Follow-Up Attendance **eTable 10.** Percentage of Follow-Up by Maternal Education for Each Neurodevelopmental Assessment Comparing AGA and FGR **eTable 11.** Follow-Up by Maternal Education for Each Neurodevelopmental Assessment **eFigure 1.** Correlations Between Fetal Circulatory Parameters and Fetal Growth **eFigure 2.** Correlations Between Fetal Hemodynamics and Perinatal Brain Growth **eFigure 3.** Correlations Between Fetal Hemodynamics and Somatic Growth **eFigure 4.** Correlations Between Fetal Hemodynamics and Neurodevelopmental Outcomes **eFigure 5.** Correlations Between Perinatal Brain Growth and Neurodevelopmental Outcomes **eFigure 6.** Correlations Between Somatic Growth and Neurodevelopmental Outcomes
eReferences.

Supplement 2.
Data Sharing Statement

## References

[1] Melamed N, Baschat A, Yinon Y, . FIGO (International Federation of Gynecology and Obstetrics) initiative on fetal growth: best practice advice for screening, diagnosis, and management of fetal growth restriction. Int J Gynaecol Obstet. 2021;152(suppl 1):3-57. doi:10.1002/ijgo.13522 33740264 PMC8252743

[2] Fetal growth restriction: ACOG practice bulletin, number 227. Obstet Gynecol. 2021;137(2):e16-e28. doi:10.1097/AOG.0000000000004251 33481528

[3] Murray E, Fernandes M, Fazel M, Kennedy SH, Villar J, Stein A. Differential effect of intrauterine growth restriction on childhood neurodevelopment: a systematic review. BJOG. 2015;122(8):1062-1072. doi:10.1111/1471-0528.13435 25990812

[4] Sacchi C, Marino C, Nosarti C, Vieno A, Visentin S, Simonelli A. Association of intrauterine growth restriction and small for gestational age status with childhood cognitive outcomes: a systematic review and meta-analysis. JAMA Pediatr. 2020;174(8):772-781. doi:10.1001/jamapediatrics.2020.1097 32453414 PMC7251506

[5] Levine TA, Grunau RE, McAuliffe FM, Pinnamaneni R, Foran A, Alderdice FA. Early childhood neurodevelopment after intrauterine growth restriction: a systematic review. Pediatrics. 2015;135(1):126-141. doi:10.1542/peds.2014-1143 25548332

[6] Martins JG, Biggio JR, Abuhamad A; Society for Maternal-Fetal Medicine (SMFM). Society for Maternal-Fetal Medicine consult series #52: diagnosis and management of fetal growth restriction. Am J Obstet Gynecol. 2020;223(4):B2-B17. doi:10.1016/j.ajog.2020.05.010 32407785

[7] Gordijn SJ, Beune IM, Thilaganathan B, . Consensus definition of fetal growth restriction: a Delphi procedure. Ultrasound Obstet Gynecol. 2016;48(3):333-339. doi:10.1002/uog.15884 26909664

[8] Fantasia I, Zamagni G, Lees C, . Current practice in the diagnosis and management of fetal growth restriction: an international survey. Acta Obstet Gynecol Scand. 2022;101(12):1431-1439. doi:10.1111/aogs.14466 36214456 PMC9812103

[9] Lees CC, Stampalija T, Baschat A, . ISUOG practice guidelines: diagnosis and management of small-for-gestational-age fetus and fetal growth restriction. Ultrasound Obstet Gynecol. 2020;56(2):298-312. doi:10.1002/uog.22134 32738107

[10] Kingdom J, Ashwal E, Lausman A, . Guideline No. 442: fetal growth restriction: screening, diagnosis, and management in singleton pregnancies. J Obstet Gynaecol Can. 2023;45(10):102154. doi:10.1016/j.jogc.2023.05.022 37730302

[11] van Wyk L, Boers KE, van Wassenaer-Leemhuis AG, ; DIGITAT Study Group. Postnatal catch-up growth after suspected fetal growth restriction at term. Front Endocrinol (Lausanne). 2019;10:274. doi:10.3389/fendo.2019.00274 31293512 PMC6598620

[12] Stoll BJ, Hansen NI, Bell EF, ; Eunice Kennedy Shriver National Institute of Child Health and Human Development Neonatal Research Network. Trends in care practices, morbidity, and mortality of extremely preterm neonates, 1993-2012. JAMA. 2015;314(10):1039-1051. doi:10.1001/jama.2015.10244 26348753 PMC4787615

[13] Vijgen SMC, Boers KE, Opmeer BC, . Economic analysis comparing induction of labour and expectant management for intrauterine growth restriction at term (DIGITAT trial). Eur J Obstet Gynecol Reprod Biol. 2013;170(2):358-363. doi:10.1016/j.ejogrb.2013.07.017 23910171

[14] Good Clinical Practice Network. ICS harmonized guideline integrated addendum to ICH E6(R1): Guideline For Good Clinical Practice E6(R2). Accessed November 9, 2016. https://ichgcp.net/

[15] World Medical Association. World Medical Association Declaration of Helsinki: ethical principles for medical research involving human subjects. JAMA. 2013;310(20):2191-2194. doi:10.1001/jama.2013.281053 24141714

[16] Zhu MY, Milligan N, Keating S, . The hemodynamics of late-onset intrauterine growth restriction by MRI. Am J Obstet Gynecol. 2016;214(3):367.e1-367.e17. doi:10.1016/j.ajog.2015.10.004 26475425

[17] Kingdom JC, Audette MC, Hobson SR, Windrim RC, Morgen E. A placenta clinic approach to the diagnosis and management of fetal growth restriction. Am J Obstet Gynecol. 2018;218(2S):S803-S817. doi:10.1016/j.ajog.2017.11.575 29254754

[18] Bhide A, Acharya G, Baschat A, . ISUOG practice guidelines (updated): use of Doppler velocimetry in obstetrics. Ultrasound Obstet Gynecol. 2021;58(2):331-339. doi:10.1002/uog.23698 34278615

[19] Fetal Medicine Foundation. Fetal Doppler z-score calculator. Accessed September 1, 2024. https://fetalmedicine.org/research/doppler.

[20] Sun L, Macgowan CK, Sled JG, . Reduced fetal cerebral oxygen consumption is associated with smaller brain size in fetuses with congenital heart disease. Circulation. 2015;131(15):1313-1323. doi:10.1161/CIRCULATIONAHA.114.013051 25762062 PMC4398654

[21] Fogel MA, Durning S, Wernovsky G, Pollock AN, Gaynor JW, Nicolson S. Brain versus lung: hierarchy of feedback loops in single-ventricle patients with superior cavopulmonary connection. Circulation. 2004;110(11)(suppl 1):II147-II152. doi:10.1161/01.CIR.0000138346.34596.99 15364854

[22] Roelfsema NM, Hop WCJ, Boito SME, Wladimiroff JW. Three-dimensional sonographic measurement of normal fetal brain volume during the second half of pregnancy. Am J Obstet Gynecol. 2004;190(1):275-280. doi:10.1016/S0002-9378(03)00911-6 14749673

[23] Archie JG, Collins JS, Lebel RR. Quantitative standards for fetal and neonatal autopsy. Am J Clin Pathol. 2006;126(2):256-265. doi:10.1309/FK9D5WBA1UEPT5BB 16891202

[24] Piper MC, Pinnell LE, Darrah J, Maguire T, Byrne PJ. Construction and validation of the Alberta Infant Motor Scale (AIMS). Can J Public Health. 1992;83(suppl 2):S46-S50. 1468050

[25] Bayley N. Bayley Scales of Infant and Toddler Development. 3rd ed. APA Psyctests; 2012.

[26] Dietitians of Canada. WHO growth charts for Canada. 2014. Accessed Month/day/year. https://www.dietitians.ca/Secondary-Pages/Public/Who-Growth-Charts.aspx

[27] Morris RK, Johnstone E, Lees C, Morton V, Smith G; Royal College of Obstetricians and Gynaecologists. Investigation and care of a small-for-gestational-age fetus and a growth restricted fetus (Green-top Guideline No. 31). BJOG. 2024;131(9):e31-e80. doi:10.1111/1471-0528.17814 38740546

[28] Sovio U, White IR, Dacey A, Pasupathy D, Smith GCS. Screening for fetal growth restriction with universal third trimester ultrasonography in nulliparous women in the Pregnancy Outcome Prediction (POP) study: a prospective cohort study. Lancet. 2015;386(10008):2089-2097. doi:10.1016/S0140-6736(15)00131-2 26360240 PMC4655320

[29] American College of Obstetricians and Gynecologists’ Committee on Obstetric Practice, Society for Maternal-Fetal Medicine. Medically indicated late-preterm and early-term deliveries: ACOG committee opinion, number 831. Obstet Gynecol. 2021;138(1):e35-e39. doi:10.1097/AOG.0000000000004447 34259491

[30] Lees CC, Romero R, Stampalija T, . Clinical opinion: the diagnosis and management of suspected fetal growth restriction: an evidence-based approach. Am J Obstet Gynecol. 2022;226(3):366-378. doi:10.1016/j.ajog.2021.11.1357 35026129 PMC9125563

[31] Meher S, Hernandez-Andrade E, Basheer SN, Lees C. Impact of cerebral redistribution on neurodevelopmental outcome in small-for-gestational-age or growth-restricted babies: a systematic review. Ultrasound Obstet Gynecol. 2015;46(4):398-404. doi:10.1002/uog.14818 25683973

[32] Giussani DA. The fetal brain sparing response to hypoxia: physiological mechanisms. J Physiol. 2016;594(5):1215-1230. doi:10.1113/JP271099 26496004 PMC4721497

[33] Selvanathan T, Miller SP. Factors affecting brain maturation trajectories in early childhood. Lancet Neurol. 2024;23(5):456-458. doi:10.1016/S1474-4422(24)00089-9 38631757

[34] Unterscheider J, O’Donoghue K, Daly S, . Fetal growth restriction and the risk of perinatal mortality—case studies from the multicentre PORTO study. BMC Pregnancy Childbirth. 2014;14:63. doi:10.1186/1471-2393-14-63 24517273 PMC3923738

[35] Rial-Crestelo M, Lubusky M, Parra-Cordero M, ; RATIO37 Study Group. Term planned delivery based on fetal growth assessment with or without the cerebroplacental ratio in low-risk pregnancies (RATIO37): an international, multicentre, open-label, randomised controlled trial. Lancet. 2024;403(10426):545-553. doi:10.1016/S0140-6736(23)02228-6 38219773

[36] Black MM, Walker SP, Fernald LCH, ; Lancet Early Childhood Development Series Steering Committee. Early childhood development coming of age: science through the life course. Lancet. 2017;389(10064):77-90. doi:10.1016/S0140-6736(16)31389-7 27717614 PMC5884058

[37] Anyigbo C, Liu C, Ehrlich S, Reyner A, Ammerman RT, Kahn RS. Household health-related social needs in newborns and infant behavioral functioning at 6 months. JAMA Pediatr. 2024;178(2):160-167. doi:10.1001/jamapediatrics.2023.5721 38147349 PMC10751658

[38] Jensen A, Garnier Y, Berger R. Dynamics of fetal circulatory responses to hypoxia and asphyxia. Eur J Obstet Gynecol Reprod Biol. 1999;84(2):155-172. doi:10.1016/S0301-2115(98)00325-X 10428339

