# Supplementary material for: Neurodevelopment Among Infants With Late-Onset Fetal Growth Restriction
Source: JAMA Netw Open. 2025 Jun 25;8(6):e2517360. doi: 10.1001/jamanetworkopen.2025.17360 (PMC12199057; doi:10.1001/jamanetworkopen.2025.17360)
Supplement: Supplement 1. — eMethods. Additional Details eTable 1. Additional Fetal Hemodynamic Parameters eTable 2. Neonatal Brain MRI Outcomes eTable 3. Comparison of Somatic Growth Trajectories Between Those of Term Infants With and Without FGR eTable 4. Neurodevelopmental Outcomes eTable 5. Sensitivity Analysis of 4-Month Follow-Up Attendance eTable 6. Sensitivity Analysis of 8-Month Follow-Up Attendance eTable 7. Sensitivity Analysis of 12-Month Follow-Up Attendance eTable 8. Sensitivity Analysis of 18-Month Follow-Up Attendance eTable 9. Sensitivity Analysis of 36-Month Follow-Up Attendance eTable 10. Percentage of Follow-Up by Maternal Education for Each Neurodevelopmental Assessment Comparing AGA and FGR eTable 11. Follow-Up by Maternal Education for Each Neurodevelopmental Assessment eFigure 1. Correlations Between Fetal Circulatory Parameters and Fetal Growth eFigure 2. Correlations Between Fetal Hemodynamics and Perinatal Brain Growth eFigure 3. Correlations Between Fetal Hemodynamics and Somatic Growth eFigure 4. Correlations Between Fetal Hemodynamics and Neurodevelopmental Outcomes eFigure 5. Correlations Between Perinatal Brain Growth and Neurodevelopmental Outcomes eFigure 6. Correlations Between Somatic Growth and Neurodevelopmental Outcomes eReferences. [file jamanetwopen-e2517360-s001.pdf]

Supplementary Online Content

Sun L, Lee F-T, Milligan N, et al. Neurodevelopment among infants with late-onset fetal growth restriction. *JAMA Netw Open.* 2025;8(6):e2517360. doi:10.1001/jamanetworkopen.2025.17360

eMethods. Additional Details
eTable 1. Additional Fetal Hemodynamic Parameters
eTable 2. Neonatal Brain MRI Outcomes
eTable 3. Comparison of Somatic Growth Trajectories Between Those of Term Infants With and Without FGR
eTable 4. Neurodevelopmental Outcomes
eTable 5. Sensitivity Analysis of 4-Month Follow-Up Attendance
eTable 6. Sensitivity Analysis of 8-Month Follow-Up Attendance
eTable 7. Sensitivity Analysis of 12-Month Follow-Up Attendance
eTable 8. Sensitivity Analysis of 18-Month Follow-Up Attendance
eTable 9. Sensitivity Analysis of 36-Month Follow-Up Attendance
eTable 10. Percentage of Follow-Up by Maternal Education for Each Neurodevelopmental Assessment Comparing AGA and FGR
eTable 11. Follow-Up by Maternal Education for Each Neurodevelopmental Assessment
eFigure 1. Correlations Between Fetal Circulatory Parameters and Fetal Growth
eFigure 2. Correlations Between Fetal Hemodynamics and Perinatal Brain Growth
eFigure 3. Correlations Between Fetal Hemodynamics and Somatic Growth
eFigure 4. Correlations Between Fetal Hemodynamics and Neurodevelopmental Outcomes
eFigure 5. Correlations Between Perinatal Brain Growth and Neurodevelopmental Outcomes
eFigure 6. Correlations Between Somatic Growth and Neurodevelopmental Outcomes
eReferences.

This supplementary material has been provided by the authors to give readers additional information about their work.

© 2025 Sun L et al. *JAMA Network Open*.

eMethods. Additional Details

Fetal Cardiovascular Magnetic Resonance

Fetal cardiovascular magnetic resonance was performed according to our previously published protocol[1] on a 1.5 T Avanto^{FIT} MR system (Siemens, Erlangen, Germany) without contrast agents or sedation at the Hospital for Sick Children. The target gestational age of assessment was 36 weeks.

Fetal brain and body weight quantification

A 3-dimensional balanced steady-state free-precession breath-hold acquisition sequence was used to obtain a 3-dimensional acquisition of the fetal body. Manual segmentation of the fetal envelope was used to obtain fetal volume and brain using a commercial postprocessing software package (Mimics, Materialize, Leuven, Belgium). Fetal body volumes were converted to estimated fetal weights based on a previously reported linear relationship between birthweight and fetal volume measured using echo-planar MRI:[2] estimated fetal weight (g) = 0.12 + 1.031 x fetal volume (mL); and converted to Z-scores based on sex-specific fetal growth charts.[3] Fetal brain volumes were converted to estimated fetal brain weights based on the assumption of a brain-specific gravity of 1.04: estimated fetal brain weight (g) = brain volume (mL) x 1.03;[4] and converted to Z-scores based on a previously published fetal and neonatal autopsy series.[5]

Fetal blood flow quantification

Phase contrast MRI was acquired and processed using metric optimized gating (github.com/MetricOptimizedGating/MOG-Public, SHA-1: 1b420a6) to quantify vessel blood flow in the following fetal vessels: ascending aorta, main pulmonary artery, left and right pulmonary arteries, superior vena cava, descending aorta, and umbilical vein. Pulmonary blood flow was derived as the sum of left and right pulmonary artery flows, ductus arteriosus flow was derived as the difference between main pulmonary artery and branch pulmonary artery flows, foramen ovale flow was derived as the difference between the ascending aorta and branch pulmonary arteries, and combined ventricular output was derived as the sum of main pulmonary artery and ascending aorta flow divided by 0.97, based on the assumption that coronary artery flow was 3% of the combined ventricular output.[6] All flow measurements were indexed to estimated fetal weight.

Fetal oximetry quantification

A T2-prepared steady-state free precession sequence with a non-rigid registration motion correction algorithm (Myomaps, Siemens Healthcare, Erlangen, Germany) was used to obtain T2 estimates of blood in the umbilical vein, superior vena cava, ascending aorta, main pulmonary artery, and descending aorta. T2 estimation was performed by placing a region of interest at the centre of the vessel with approximately 50% coverage to obtain T2 relaxation times of the major fetal vessels. The combination of vessel T2 relaxation times and gestational age specific reference values for hematocrit[7] were used to obtain vessel oxygen saturations based on a previously derived fit of oxygen saturation to *in vitro* umbilical cord human blood T2.[1,8]

Fetal oxygen transportation calculations

Fetal vessel blood flow, oxygen saturations and gestational age-appropriate population averages of hemoglobin concentrations ([Hb]) were used to calculate fetal oxygen transportation variables.[7]

Fetal oxygen delivery = umbilical vein flow x umbilical vein oxygen saturation x [Hb]
Fetal oxygen consumption = umbilical vein flow x (umbilical vein – descending aorta saturation) x [Hb]
Fetal oxygen extraction = fetal oxygen consumption/fetal oxygen delivery
Fetal cerebral oxygen delivery = superior vena cava flow x ascending aortic oxygen saturation x [Hb]
Fetal cerebral oxygen consumption = superior vena cava flow x (ascending aortic – superior vena caval oxygen saturation) x [Hb]
Fetal cerebral oxygen extraction = fetal cerebral oxygen consumption/fetal cerebral oxygen delivery
Cerebral oxygen delivery to fetal oxygen delivery ratio = fetal cerebral oxygen delivery/fetal oxygen delivery

Descending aortic oxygen saturations were used as a surrogate for umbilical artery oxygen saturations as the umbilical arteries branches of the internal iliac arteries which arise from the common iliac arteries, which arise from the descending aorta.

© 2025 Sun L et al. *JAMA Network Open*.

Neonatal Brain Magnetic Resonance Imaging

Neonatal brain MRI was performed on a 1.5 T Avanto^{FIT} MR system (Siemens, Erlangen, Germany) without contrast agents or sedation. Subjects were scanned using an unsedated "feed-and-sleep" approach.[9]

Neonatal brain weight quantification

A 3-dimensional balanced steady-state free-precession sequence was used to obtain 3-dimensional images of the neonatal brain. Manual segmentation was performed to obtain volumes of the neonatal brain using a postprocessing commercial software package (Mimics, Materialize, Leuven, Belgium). Neonatal brain volumes were converted to estimated neonatal brain weights based on the assumption of a brain-specific gravity of 1.04: estimated fetal brain weight (g) = brain volume (mL) x 1.03;[4] and were converted to Z-scores based on a previously published fetal and neonatal autopsy series.[5]

Neonatal diffusion weighted imaging

Diffusion weighted imaging was used to obtain the apparent diffusion coefficient (ADC) in predefined regions of interest in the neonatal brain according to our previously published protocol:[10]
Anterior white matter (left, right, and averaged)
Central white matter (left, right, and averaged)
Posterior white matter (left, right, and averaged)
Genu of the corpus callosum (left, right, and averaged)
Splenium of the corpus callosum (left, right, and averaged)
Posterior limp of the internal capsule (left, right, and averaged)
Optic radiations (left, right, and averaged)
Caudate (left, right, and averaged)
Lentiform nucleus (left, right, and averaged)
Thalamus (left, right, and averaged)
Calcarine region (left, right, and averaged)
Anterior white matter at the level of the basal ganglia (left, right, and averaged)
Posterior white matter at the ventricular level (left, right, and averaged)
Basal ganglia = average of caudate, lentiform nucleus, and thalamus
Global white matter = average of the anterior, central, and posterior white matter, anterior white matter at the basal ganglia level, and posterior white matter at the ventricular level
Corpus callosum = average of the genu and splenium

Neonatal diffusion tensor imaging

Diffusion tensor imaging was used to obtain the fractional anisotropy (FA) in predefined regions of interest in the neonatal brain as per previously published protocol:[10]
Anterior white matter (left, right, and averaged)
Central white matter (left, right, and averaged)
Posterior white matter (left, right, and averaged)
Genu of the corpus callosum (left, right, and averaged)
Splenium of the corpus callosum (left, right, and averaged)
Posterior limp of the internal capsule (left, right, and averaged)
Optic radiations (left, right, and averaged)
Caudate (left, right, and averaged)
Lentiform nucleus (left, right, and averaged)
Thalamus (left, right, and averaged)
Calcarine region (left, right, and averaged)
Anterior white matter at the level of the basal ganglia (left, right, and averaged)
Posterior white matter at the ventricular level (left, right, and averaged)
Basal ganglia = average of caudate, lentiform nucleus, and thalamus
Global white matter = average of the anterior, central, and posterior white matter, anterior white matter at the basal ganglia level, and posterior white matter at the ventricular level
Corpus callosum = average of the genu and splenium

Neonatal magnetic resonance spectroscopy

© 2025 Sun L et al. *JAMA Network Open*.

Magnetic resonance spectroscopy was obtained using multivoxel chemical shift imaging. Sixteen predefined regions of interest were placed at 2 different levels of the neonatal brain: (1) high centrum semiovale above the lateral ventricles, and (2) basal ganglia at the level of the foramen of Monro as per previously published protocol.[11] The average of the lactate, N-acetyl aspartate to choline ratios, choline to creatine ratios, and N-acetyl aspartate to creatine ratios were reported.

© 2025 Sun L et al. *JAMA Network Open*.

eTable 1. Additional Fetal Hemodynamic Parameters

	Mean (SD)				Preterm FGR vs. Term FGR		Preterm FGR vs. AGA		Term FGR vs. AGA	
**Parameters**	**Preterm FGR**	**Term FGR**	**AGA**	**P value**	**Mean Difference (95% CI)**	**P value**	**Mean Difference (95% CI)**	**P value**	**Mean Difference (95% CI)**	**P value**
**Main pulmonary artery flow to combined ventricular output ratio**	57.9 (10.4)	55.7 (7.9)	53.7 (7.6)	0.18	2.2 (-3.1 to 7.5)	0.41	4.2 (-0.5 to 8.9)	0.08	2.0 (-1.9 to 6.0)	0.31
**Ascending aorta flow to combined ventricular output ratio**	37.5 (9.1)	41.5 (8.0)	44.2 (7.8)	0.01	-4.0 (-9.2 to 1.2)	0.13	-6.8 (-11.4 to -2.0)	0.005	-2.8 (-6.7 to 1.1)	0.16
**Superior vena cava flow to combined ventricular output ratio**	44.2 (10.4)	32.2 (8.9)	29.2 (8.5)	<0.001	12.0 (6.1 to 17.8)	<0.001	15 (9.8 to 20.2)	<0.001	3.0 (-1.3 to 7.3)	0.17
**Ductus arteriosus flow to combined ventricular output ratio**	47.8 (13.2)	41.5 (10.0)	39.1 (8.4)	0.01	6.3 (-0.2 to 12.8)	0.06	8.7 (2.9 to 14.5)	0.003	2.4 (-2.4 to 7.2)	0.32
**Descending aorta flow to combined ventricular output ratio**	45.9 (10.5)	51.6 (8.9)	54.7 (9.5)	0.007	-5.7 (-11.8 to 0.5)	0.07	-8.8 (-14.2 to -3.2)	0.002	-3.1 (-7.7 to 1.5)	0.18
**Pulmonary blood flow to combined ventricular output ratio**	9.7 (5.7)	13.2 (6.5)	15.6 (7.0)	0.03	-3.5 (-8.6 to 1.5)	0.17	-5.9 (-10.4 to -1.2)	0.01	-2.3 (-5.7 to 1.0)	0.17
**Umbilical vein flow to combined ventricular output ratio**	17.8 (5.3)	27.9 (7.0)	27.7 (6.5)	<0.001	-10.1 (-14.4 to -5.7)	<0.001	-9.9 (-13.8 to -6.0)	<0.001	0.2 (-3.0 to 3.3)	0.92

Ratios are expressed as a proportion out of 100. FGR, fetal growth restriction; AGA, appropriate-for-gestation age

© 2025 Sun L et al. *JAMA Network Open*.

eTable 2. Neonatal Brain MRI Outcomes

	n	AGA	n	Term FGR	Mean Difference (95% CI)	P value
**Neonatal brain weight Z-Score**	38	0.37 (0.87)	15	-0.07 (0.67)	-0.45 (-0.96 to 0.05)	0 .08
**Apparent Diffusion Coefficient**						
**Anterior white matter**	33	1565 (176)	13	1587 (194)	22 (-97 to 141)	0.71
**Central white matter**	33	1416 (136)	13	1458 (210)	42 (-63 to 147)	0.42
**Posterior white matter**	33	1469 (203)	13	1497 (190)	27 (-104 to 159)	0.67
**Genu of the corpus callous**	33	1191 (154)	13	1234 (213)	42 (-70 to 156)	0.44
**Splenium of the corpus callous**	33	1201 (264)	13	1061 (176)	-140 (-301 to 19)	0.08
**Posterior limb of internal capsule**	33	1038 (101)	13	1035 (91)	-2 (-67 to 62)	0.93
**Optic radiations**	33	1374 (144)	13	1434 (199)	59 (-46 to 165)	0.26
**Caudate**	33	1188 (65)	13	1209 (57)	20 (-20 to 61)	0.32
**Lentiform nucleus**	33	1120 (64)	13	1124 (50)	4 (-35 to 44)	0.82
**Thalamus**	33	1082 (52)	13	1093 (59)	11 (-24 to 46)	0.52
**Calcarine region**	33	1299 (94)	13	1273 (90)	-25 (-87 to 35)	0.39
**Anterior white matter at basal ganglia level**	33	1672 (161)	13	1708 (192)	36 (-75 to 149)	0.51
**Posterior white matter at ventricular level**	33	1738 (183)	13	1766 (144)	28 (-86 to 142)	0.62
**Global white matter**	28	1494 (138)	10	1506 (205)	11 (-106 to 129)	0.84
**Basal ganglia**	28	1131 (51)	10	1148 (32)	17 (-177 to 52)	0.32
**Corpus callosum**	28	1173 (157)	10	1142 (151)	-31 (-147 to 85)	0.59
**Fractional Anisotropy**						
**Anterior white matter**	33	0.17 (0.04)	13	0.16 (0.05)	-0.01 (-0.03 to 0.02)	0.75
**Central white matter**	33	0.22 (0.04)	13	0.22 (0.06)	0 (-0.03 to 0.03)	0.82
**Posterior white matter**	33	0.21 (0.05)	13	0.21 (0.06)	0 (-0.04 to 0.03)	0.78
**Genu of the corpus callous**	33	0.72 (0.07)	13	0.71 (0.11)	-0.01 (-0.06 to 0.04)	0.70
**Splenium of the corpus callous**	33	0.75 (0.09)	13	0.77 (0.08)	0.03 (-0.03 to 0.08)	0.39

© 2025 Sun L et al. *JAMA Network Open*.

	n	AGA	n	Term FGR	Mean Difference (95% CI)	P value
**Posterior limb of internal capsule**	33	0.61 (0.07)	13	0.61 (0.05)	0.01 (-0.04 to 0.05)	0.78
**Optic radiations**	33	0.37 (0.06)	13	0.40 (0.10)	0.04 (-0.01 to 0.08)	0.18
**Caudate**	33	0.14 (0.03)	13	0.15 (0.04)	0.01 (-0.01 to 0.03)	0.28
**Lentiform nucleus**	33	0.17 (0.03)	13	0.18 (0.04)	0.01 (-0.01 to 0.03)	0.37
**Thalamus**	33	0.17 (0.03)	13	0.20 (0.04)	0.03 (0.00 to 0.04)	0.03
**Calcarine region**	33	0.21 (0.04)	13	0.21 (0.05)	0 (-0.03 to 0.02)	0.82
**Anterior white matter at basal ganglia level**	33	0.15 (0.04)	13	0.17 (0.07)	0.02 (-0.01 to 0.05)	0.29
**Posterior white matter at ventricular level**	33	0.16 (0.04)	13	0.16 (0.04)	-0.01 (-0.03 to 0.02)	0.70
**Global white matter**	33	0.16 (0.03)	13	0.18 (0.03)	0.02 (-0.00 to 0.03)	0.13
**Basal ganglia**	33	0.18 (0.03)	13	0.18 (0.05)	0 (-0.02 to 0.02)	0.98
**Corpus callosum**	33	0.73 (0.07)	13	0.74 (0.08)	0.01 (-0.04 to 0.05)	0.75
**Cerebral Metabolites**						
**Lactate**	35	7/35 (20)	13	5/13 (38)	n/a	0.26
**NAA: Cho**	35	0.63 (0.12)	13	0.62 (0.13)	-0.01 (-0.09 to 0.07)	0.84
**Cho: Creatine**	34	1.97 (0.31)	13	1.86 (0.37)	-0.11 (-0.32 to 0.11)	0.33
**NAA: Creatine**	35	1.22 (0.19)	13	1.12 (0.19)	-0.1 (-0.22 to 0.02)	0.11

FGR, fetal growth restriction; AGA, appropriate-for-gestation age

© 2025 Sun L et al. *JAMA Network Open*.

eTable 3. Comparison of Somatic Growth Trajectories Between Those of Term Infants With and Without FGR

	n	AGA	n	Term FGR	Mean Difference (95% CI)	P value
**Weight Z-score**						
Birth	35	0.02	15	-1.60	-1.62 (-2.31 to -0.93)	<0.001
4 months	31	-0.06	12	-1.15	-1.09 (-2.09 to -0.09)	0.03
8 months	31	0.31	12	-0.82	-1.13 (-2.33 to 0.06)	0.07
12 months	29	0.06	14	-0.45	-0.51 (-1.37 to 0.36)	0.49
18 months	32	0.49	14	0.05	-0.44 (-1.32 to 0.44)	0.67
36 months	25	0.16	14	-0.22	-0.38 (-1.29 to 0.53)	0.81
**Length Z-score**						
Birth	35	1.41	15	-0.63	-2.04 (-3.22 to -0.85)	<0.001
4 months	32	0.38	12	-0.88	-1.27 (-2.13 to -0.40)	0.002
8 months	32	0.20	12	-0.49	-0.68 (-1.56 to 0.20)	0.19
12 months	29	-0.01	14	-0.37	-0.36 (-1.27 to 0.56)	0.87
18 months	32	-0.40	12	-0.91	-0.51 (-1.32 to 0.30)	0.40
36 months	25	-0.34	14	-0.47	-0.14 (-1.15 to 0.87)	1.00
**BMI Z-score**						
Birth	34	-1.03	16	-2.14	-1.11 (-1.87 to -0.35)	0.002
4 months	31	-0.38	13	-0.79	-0.40 (-1.26 to 0.45)	0.71
8 months	31	0.27	12	-0.73	-0.99 (-2.08 to 0.09)	0.08
12 months	29	0.10	14	-0.32	-0.42 (-1.28 to 0.44)	0.69
18 months	32	1.02	11	0.76	-0.26 (-1.29 to 0.77)	0.97
36 months	25	0.53	14	0.09	-0.44 (-1.31 to 0.43)	0.66
**Head Circumference Z-score**						
Birth	35	0.26	15	-1.02	-1.29 (-2.11 to -0.46)	<0.001
4 months	32	0.51	12	-0.64	-1.14 (-1.86 to -0.43)	<0.001
8 months	32	0.53	12	-0.33	-0.86 (-1.58 to -0.14)	0.01
12 months	29	0.53	14	-0.49	-1.02 (-1.67 to -0.36)	<0.001
18 months	32	0.72	15	-0.04	-0.77 (-1.47 to -0.07)	0.03
36 months	25	0.82	14	-0.13	-0.95 (-1.74 to -0.17)	0.01

FGR, fetal growth restriction; AGA, appropriate-for-gestation age; BMI, body mass index.

© 2025 Sun L et al. *JAMA Network Open*.

eTable 4. Neurodevelopmental Outcomes

	n	AGA	n	Term FGR	Mean difference (95% CI)	P value
**4 month AIMS**						
**Total score**	34	16.1 (4.6)	14	14.8 (2.2)	-1.3 (-3.8 to 1.2)	0.31
**8 month AIMS**						
**Total score**	33	39.2 (8.2)	12	36.3 (11.8)	-2.99 (-9.2 to 3.3)	0.34
**12 month AIMS**						
**Total score**	28	53.7 (4.9)	14	49.1 (8.5)	-4.5 (-8.6 to -0.3)	0.03
**18 month BSID-III**						
**Cognitive Composite Scores**	31	107.1 (13.1)	15	100.7 (13.6)	-6.4 (-14.8 to 1.9)	0.12
**Language Composite Scores**	31	108 (18.4)	15	96.6 (25.6)	-11.3 (-24.6 to 1.8)	0.09
**Motor Composite Scores**	30	104.5 (9.9)	15	101.1 (14.5)	-3.3 (-10.6 to 3.9)	0.36
**36 month BSID-III**						
**Cognitive Composite Scores**	25	104.8 (9)	14	102.9 (11.9)	-1.9 (-8.7 to 4.8)	0.56
**Language Composite Scores**	22	113.2 (11.2)	13	106.9 (13.7)	-6.3 (-14.9 to 2.3)	0.14

FGR, fetal growth restriction; AGA, appropriate-for-gestation age; AIMS, Alberta Infant Motor Scale; BSID-III, Bayley Scales of Infant and Toddler Development 3rd Edition.

© 2025 Sun L et al. *JAMA Network Open*.

eTable 5. Sensitivity Analysis of 4-Month Follow-Up Attendance

	AGA		FGR		
**Follow-up Attendance**	**Yes**	**No**	**Yes**	**No**	**P value**
**n**	34	22	14	11	
**Maternal age at conception, years**	34.3 (3.2)	32.2 (3.4)	33.2 (5.3)	35 (4.3)	0.21
**GA at birth, weeks**	39.3 (1.1)	39.4 (1.2)	38.9 (1.2)	38.8 (1.4)	0.36
**APGAR at 1 minute**	8.8 (0.6)	8.3 (1.4)	8.7 (0.5)	8.1 (1.8)	0.16
**APGAR at 5 minutes**	9 (0)	9 (0)	9 (0)	8.9 (0.3)	0.11
**NICU admission**					**0.04**
**Yes**	4/34 (12)	1/22 (5)	2/14 (14)	2/11 (18)	
**No**	30/34 (88)	16/22 (73)	12/14 (86)	9/11 (82)	
**Missing data**	0/34 (0)	5/22 (23)	0/14 (0)	0/11 (0)	
**Maternal ethnicity/race**					0.20
**White (European, Middle Eastern, North Africa, Hispanic)**	23/34 (68)	15/22 (68)	9/14 (64)	7/11 (64)	
**East Asian (Chinese, Korea, Japanese)**	10/34 (29)	3/22 (14)	3/14 (21)	2/11 (18)	
**South Asian (Indian, Pakistani, Bangladesh)**	1/34 (3)	1/22 (5)	0	1/11 (9)	
**Black (African, Caribbean, African-American)**	0	1/22 (5)	2/14 (14)	0	
**Unreported**	0	2/22 (9)	0	1/11 (9)	
**Maternal Education**					**0.002**
**Elementary school**	0/34 (0)	0/22 (0)	0/14 (0)	0/11 (0)	
**Some high school**	0/34 (0)	0/22 (0)	0/14 (0)	0/11 (0)	
**High school diploma**	0/34 (0)	1/22 (5)	0/14 (0)	1/11 (9)	
**Some university/college**	0/34 (0)	0/22 (0)	1/14 (7)	0/11 (0)	
**University/college**	21/34 (62)	13/22 (59)	9/14 (64)	3/11 (27)	
**Graduate/professional**	8/34 (24)	0/22 (0)	4/14 (29)	2/11 (18)	
**Unreported**	5/34 (15)	8/22 (36)	0/14 (0)	5/11 (45)	

© 2025 Sun L et al. *JAMA Network Open*.

eTable 6. Sensitivity Analysis of 8-Month Follow-Up Attendance

	AGA		FGR		
**Follow-up Attendance**	**Yes**	**No**	**Yes**	**No**	**P value**
**n**					
**Maternal age at conception, years**	33.8 (3)	32.9 (3.9)	33.1 (5.3)	34.4 (4.4)	0.69
**GA at birth, weeks**	39.4 (1)	39.3 (1.3)	39 (1.2)	38.7 (1.3)	0.28
**APGAR at 1 minute**	8.6 (0.8)	8.6 (1.2)	8.8 (0.5)	8.2 (1.7)	0.48
**APGAR at 5 minutes**	9 (0)	9 (0)	9 (0)	8.9 (0.3)	0.18
**NICU admission**					0.05
**Yes**	3/33 (9)	2/23 (9)	2/12 (17)	2/13 (15)	
**No**	30/33 (91)	16/23 (70)	10/12 (83)	11/13 (85)	
**Missing data**	0/33 (0)	5/23 (22)	0/12 (0)	0/13 (0)	
**Maternal ethnicity/race**					0.18
**White (European, Middle Eastern, North Africa, Hispanic)**	25/33 (76)	13/23 (57)	7/12 (58)	9/13 (69)	
**East Asian (Chinese, Korea, Japanese)**	8/33 (24)	5/23 (22)	3/12 (25)	2/13 (15)	
**South Asian (Indian, Pakistani, Bangladesh)**	0/33 (0)	2/23 (9)	0/12 (0)	1/13 (8)	
**Black (African, Caribbean, African-American)**	0/33 (0)	1/23 (4)	2/12 (17)	0/13 (0)	
**Unreported**	0/33 (0)	2/23 (9)	0/12 (0)	1/13 (8)	
**Maternal Education**					**0.002**
**Elementary school**	0/33 (0)	0/23 (0)	0/12 (0)	0/13 (0)	
**Some high school**	0/33 (0)	0/23 (0)	0/12 (0)	0/13 (0)	
**High school diploma**	0/33 (0)	1/23 (4)	0/12 (0)	1/13 (8)	
**Some university/college**	0/33 (0)	0/23 (0)	1/12 (8)	0/13 (0)	
**University/college**	21/33 (64)	13/23 (57)	7/12 (58)	5/13 (38)	
**Graduate/professional**	8/33 (24)	0/23 (0)	4/12 (33)	2/13 (15)	
**Unreported**	4/33 (12)	9/23 (39)	0/12 (0)	5/13 (38)	

© 2025 Sun L et al. *JAMA Network Open*.

eTable 7. Sensitivity Analysis of 12-Month Follow-Up Attendance

	AGA		FGR		
**Follow-up Attendance**	**Yes**	**No**	**Yes**	**No**	**P value**
**n**	28	28	14	11	
**Maternal age at conception, years**	34.6 (3.1)	32.3 (3.3)	33.2 (5.6)	34.5 (3.7)	0.11
**GA at birth, weeks**	39.4 (1.1)	39.4 (1.2)	39.2 (1.1)	38.4 (1.3)	0.10
**APGAR at 1 minute**	8.8 (0.6)	8.5 (1.2)	8.9 (0.4)	7.9 (1.8)	0.10
**APGAR at 5 minutes**	9 (0)	9 (0)	9 (0)	8.9 (0.3)	0.11
**NICU admission**					0.06
**Yes**	2/28 (7)	3/28 (11)	1/14 (7)	3/11 (27)	
**No**	26/28 (93)	20/28 (71)	13/14 (93)	8/11 (73)	
**Missing data**	0/28 (0)	5/28 (18)	0/14 (0)	0/11 (0)	
**Maternal ethnicity/race**					0.82
**White (European, Middle Eastern, North Africa, Hispanic)**	20/28 (71)	18/28 (64)	9/14 (64)	7/11 (64)	
**East Asian (Chinese, Korea, Japanese)**	7/28 (25)	6/28 (21)	3/14 (21)	2/11 (18)	
**South Asian (Indian, Pakistani, Bangladesh)**	1/28 (4)	1/28 (4)	1/14 (7)	0/11 (0)	
**Black (African, Caribbean, African-American)**	0/28 (0)	1/28 (4)	1/14 (7)	1/11 (9)	
**Unreported**	0/28 (0)	2/28 (7)	0/14 (0)	1/11 (9)	
**Maternal Education**					**<0.001**
**Elementary school**	0/28 (0)	0/28 (0)	0/14 (0)	0/11 (0)	
**Some high school**	0/28 (0)	0/28 (0)	0/14 (0)	0/11 (0)	
**High school diploma**	0/28 (0)	1/28 (4)	0/14 (0)	1/11 (9)	
**Some university/college**	0/28 (0)	10/28 (36)	1/14 (7)	3/11 (27)	
**University/college**	18/28 (64)	16/28 (57)	6/14 (43)	6/11 (55)	
**Graduate/professional**	7/28 (25)	1/28 (4)	5/14 (36)	1/11 (9)	
**Unreported**	3/28 (11)	0/28 (0)	2/14 (14)	0/11 (0)	

© 2025 Sun L et al. *JAMA Network Open*.

eTable 8. Sensitivity Analysis of 18-Month Follow-Up Attendance

	AGA		FGR		
**Follow-up Attendance**	**Yes**	**No**	**Yes**	**No**	**P value**
**n**	31	25	15	10	
**Maternal age at conception, years**	34.1 (3.2)	32.7 (3.6)	33.6 (5.7)	34.0 (3.4)	0.58
**GA at birth, weeks**	39.4 (1.1)	39.3 (1.2)	39.1 (1.1)	38.5 (1.4)	0.16
**APGAR at 1 minute**	8.6 (0.9)	8.7 (1.1)	8.8 (0.4)	7.9 (1.9)	0.18
**APGAR at 5 minutes**	9 (0)	9 (0)	9 (0)	8.9 (0.3)	0.08
**NICU admission**					0.43
**Yes**	3/31 (10)	2/25 (8)	2/15 (13)	2/10 (20)	
**No**	27/31 (87)	19/25 (76)	13/15 (87)	8/10 (80)	
**Missing data**	1/31 (3)	4/25 (16)	0/15 (0)	0/10 (0)	
**Maternal ethnicity/race**					0.58
**White (European, Middle Eastern, North Africa, Hispanic)**	23/31 (74)	15/25 (60)	9/15 (60)	7/10 (70)	
**East Asian (Chinese, Korea, Japanese)**	7/31 (23)	6/25 (24)	3/15 (20)	2/10 (20)	
**South Asian (Indian, Pakistani, Bangladesh)**	1/31 (3)	1/25 (4)	1/15 (7)	0/10 (0)	
**Black (African, Caribbean, African-American)**	0/31 (0)	1/25 (4)	2/15 (13)	0/10 (0)	
**Unreported**	0/31 (0)	2/25 (8)	0/15 (0)	1/10 (10)	
**Maternal Education**					**0.01**
**Elementary school**	0/31 (0)	0/25 (0)	0/15 (0)	0/10 (0)	
**Some high school**	0/31 (0)	0/25 (0)	0/15 (0)	0/10 (0)	
**High school diploma**	0/31 (0)	1/25 (4)	0/15 (0)	1/10 (10)	
**Some university/college**	0/31 (0)	0/25 (0)	1/15 (7)	0/10 (0)	
**University/college**	19/31 (61)	15/25 (60)	7/15 (47)	5/10 (50)	
**Graduate/professional**	8/31 (26)	0/25 (0)	5/15 (33)	1/10 (10)	
**Unreported**	4/31 (13)	9/25 (36)	2/15 (13)	3/10 (30)	

© 2025 Sun L et al. *JAMA Network Open*.

eTable 9. Sensitivity Analysis of 36-Month Follow-Up Attendance

	AGA		FGR		
**Follow-up Attendance**	**Yes**	**No**	**Yes**	**No**	**P value**
**n**	25	31	14	11	
**Maternal age at conception, years**	35 (3.2)	32.3 (3.2)	33.1 (5.6)	34.6 (3.8)	0.05
**GA at birth, weeks**	39.3 (1.1)	39.4 (1.2)	39.2 (1.1)	38.5 (1.3)	0.14
**APGAR at 1 minute**	8.9 (0.3)	8.4 (1.2)	8.9 (0.4)	7.9 (1.8)	**0.04**
**APGAR at 5 minutes**	9 (0)	9 (0)	9 (0)	8.9 (0.3)	0.11
**NICU admission**					0.06
**Yes**	2/25 (8)	3/31 (10)	1/14 (7)	3/11 (27)	
**No**	22/25 (88)	24/31 (77)	13/14 (93)	8/11 (73)	
**Missing data**	1/25 (4)	0/31 (0)	4/14 (29)	0/11 (0)	
**Maternal ethnicity/race**					0.69
**White (European, Middle Eastern, North Africa, Hispanic)**	19/25 (76)	19/31 (61)	8/14 (57)	8/11 (73)	
**East Asian (Chinese, Korea, Japanese)**	5/25 (20)	8/31 (26)	3/14 (21)	2/11 (18)	
**South Asian (Indian, Pakistani, Bangladesh)**	1/25 (4)	1/31 (3)	1/14 (7)	0/11 (0)	
**Black (African, Caribbean, African-American)**	0/25 (0)	1/31 (3)	2/14 (14)	0/11 (0)	
**Unreported**	0/25 (0)	2/31 (6)	0/14 (0)	1/11 (9)	
**Maternal Education**					0.30
**Elementary school**	0/25 (0)	0/31 (0)	0/14 (0)	0/11 (0)	
**Some high school**	0/25 (0)	0/31 (0)	0/14 (0)	0/11 (0)	
**High school diploma**	0/25 (0)	1/31 (3)	0/14 (0)	1/11 (9)	
**Some university/college**	0/25 (0)	0/31 (0)	1/14 (7)	0/11 (0)	
**University/college**	15/25 (60)	19/31 (61)	7/14 (50)	5/11 (45)	
**Graduate/professional**	6/25 (24)	2/31 (6)	4/14 (29)	2/11 (18)	
**Unreported**	4/25 (16)	9/31 (29)	2/14 (14)	3/11 (27)	

© 2025 Sun L et al. *JAMA Network Open*.

eTable 10. Percentage of Follow-Up by Maternal Education for Each Neurodevelopmental Assessment Comparing AGA and FGR

**Timing of Assessment**	**4m**				**8m**				**12m**				**18m**				**36m**			
**Diagnosis**	**AGA**		**FGR**		**AGA**		**FGR**		**AGA**		**FGR**		**AGA**		**FGR**		**AGA**		**FGR**	
**Follow-up Attendance**	**Yes**	**No**	**Yes**	**No**	**Yes**	**No**	**Yes**	**No**	**Yes**	**No**	**Yes**	**No**	**Yes**	**No**	**Yes**	**No**	**Yes**	**No**	**Yes**	**No**
**Maternal Education**																				
**Elementary school**	NA	NA	NA	NA	NA	NA	NA	NA	NA	NA	NA	NA	NA	NA	NA	NA	NA	NA	NA	NA
**Some high school**	NA	NA	NA	NA	NA	NA	NA	NA	NA	NA	NA	NA	NA	NA	NA	NA	NA	NA	NA	NA
**High school diploma**	0 (0)	1 (100)	0 (0)	1 (100)	0 (0)	1 (100)	0 (0)	1 (100)	0 (0)	1 (100)	0 (0)	1 (100)	0 (0)	1 (100)	0 (0)	1 (100)	0 (0)	1 (100)	0 (0)	1 (100)
**Some university/college**	NA	NA	1 (100)	0 (0)	NA	NA	1 (100)	0 (0)	0 (0)	10 (100)	1 (25)	3 (75)	NA	NA	1 (100)	0 (0)	NA	NA	1 (100)	0 (0)
**University/ college**	21 (62)	13 (38)	9 (75)	3 (25)	21 (62)	13 (38)	7 (58)	5 (42)	18 (53)	16 (47)	6 (50)	6 (50)	19 (56)	15 (44)	7 (58)	5 (42)	15 (44)	19 (56)	7 (58)	5 (42)
**Graduate/ professional**	8 (100)	0 (0)	4 (67)	2 (33)	8 (100)	0 (0)	4 (67)	2 (33)	7 (88)	1 (13)	5 (83)	1 (17)	8 (100)	0 (0)	5 (83)	1 (17)	6 (75)	2 (25)	4 (67)	2 (33)

Results are presented in n (%).

© 2025 Sun L et al. *JAMA Network Open*.

eTable 11. Follow-Up by Maternal Education for Each Neurodevelopmental Assessment

Timing of Assessment	4m		8m		12m		18m		36m	
**Follow-up Attendance**	**Yes**	**No**	**Yes**	**No**	**Yes**	**No**	**Yes**	**No**	**Yes**	**No**
**Maternal Education**										
**Elementary school**	NA	NA	NA	NA	NA	NA	NA	NA	NA	NA
**Some high school**	NA	NA	NA	NA	NA	NA	NA	NA	NA	NA
**High school diploma**	0 (0)	2 (100)	0 (0)	2 (100)	0 (0)	2 (100)	0 (0)	2 (100)	0 (0)	2 (100)
**Some university/college**	1 (100)	0 (0)	1 (100)	0 (0)	1 (7)	13 (93)	1 (100)	0 (0)	1 (100)	0 (0)
**University/college**	30 (65)	16 (35)	28 (61)	18 (39)	24 (52)	22 (48)	26 (57)	20 (43)	22 (48)	24 (52)
**Graduate/professional**	12 (86)	2 (14)	12 (86)	2 (14)	12 (86)	2 (14)	13 (93)	1 (7)	10 (71)	4 (29)

Results are presented in n (%).

© 2025 Sun L et al. *JAMA Network Open*.

eFigure 1. Correlations Between Fetal Circulatory Parameters and Fetal Growth

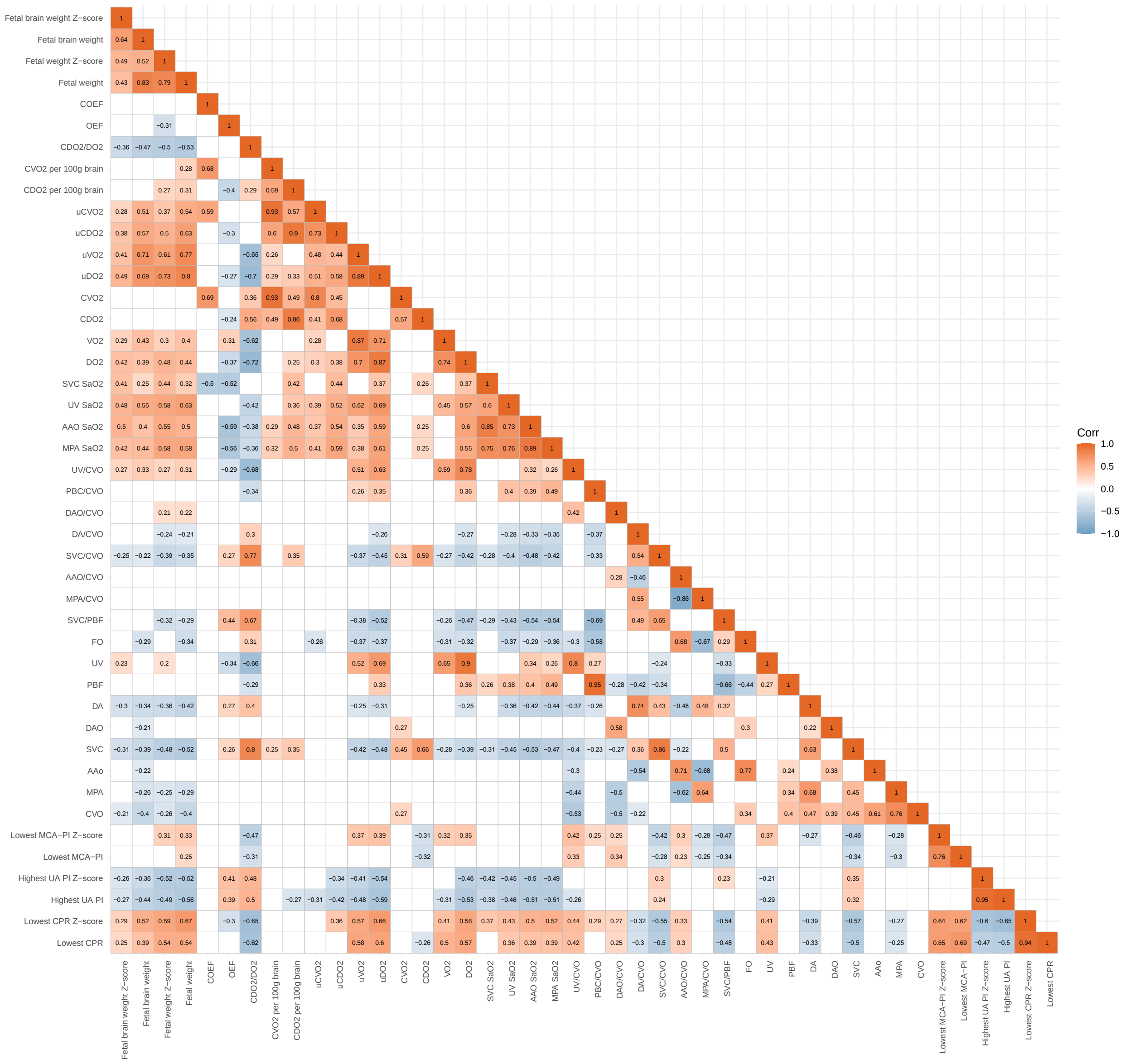

Significant correlations are shown in colour. COEF, cerebral oxygen extraction fraction; OEF, oxygen extraction fraction; CDO2, cerebral oxygen delivery; DO2, fetal oxygen delivery; u, unindexed; CVO2, cerebral oxygen consumption; CDO2, cerebral oxygen delivery; VO2, fetal oxygen consumption; DO2, fetal oxygen delivery; SaO2, oxygen saturation; SVC, superior vena cava; UV, umbilical vein; AAO, ascending aorta; MPA, main pulmonary artery; UV, umbilical vein; PBF, pulmonary blood flow; CVO, combined ventricular output; DAo, descending aorta; DA, ductus arteriosus FO, foramen ovale; MCA-PI, middle cerebral artery pulsatility index; UA PI, umbilical artery pulsatility index; CPR, cerebroplacental ratio.

© 2025 Sun L et al. *JAMA Network Open*.

eFigure 2. Correlations Between Fetal Hemodynamics and Perinatal Brain Growth
(A)

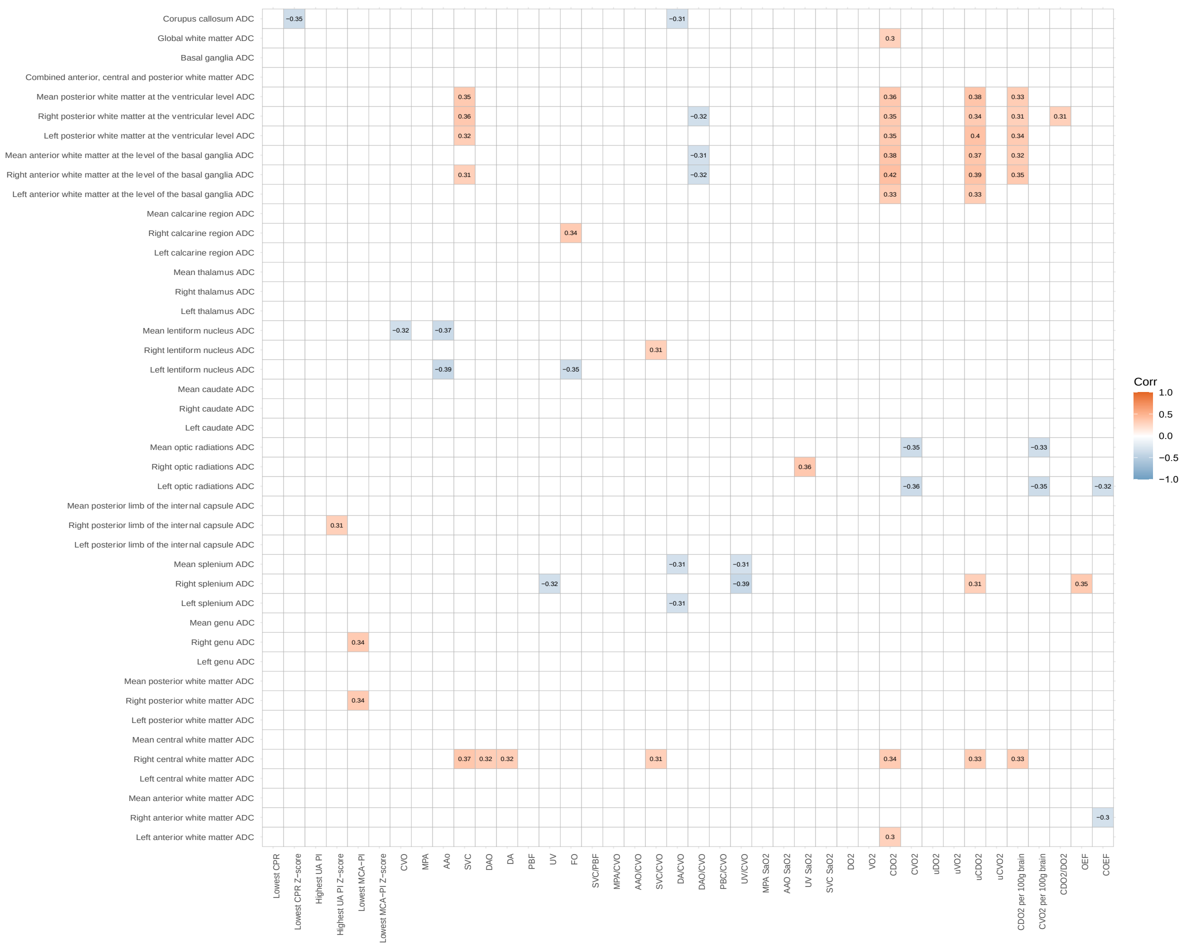

© 2025 Sun L et al. *JAMA Network Open*.

(B)

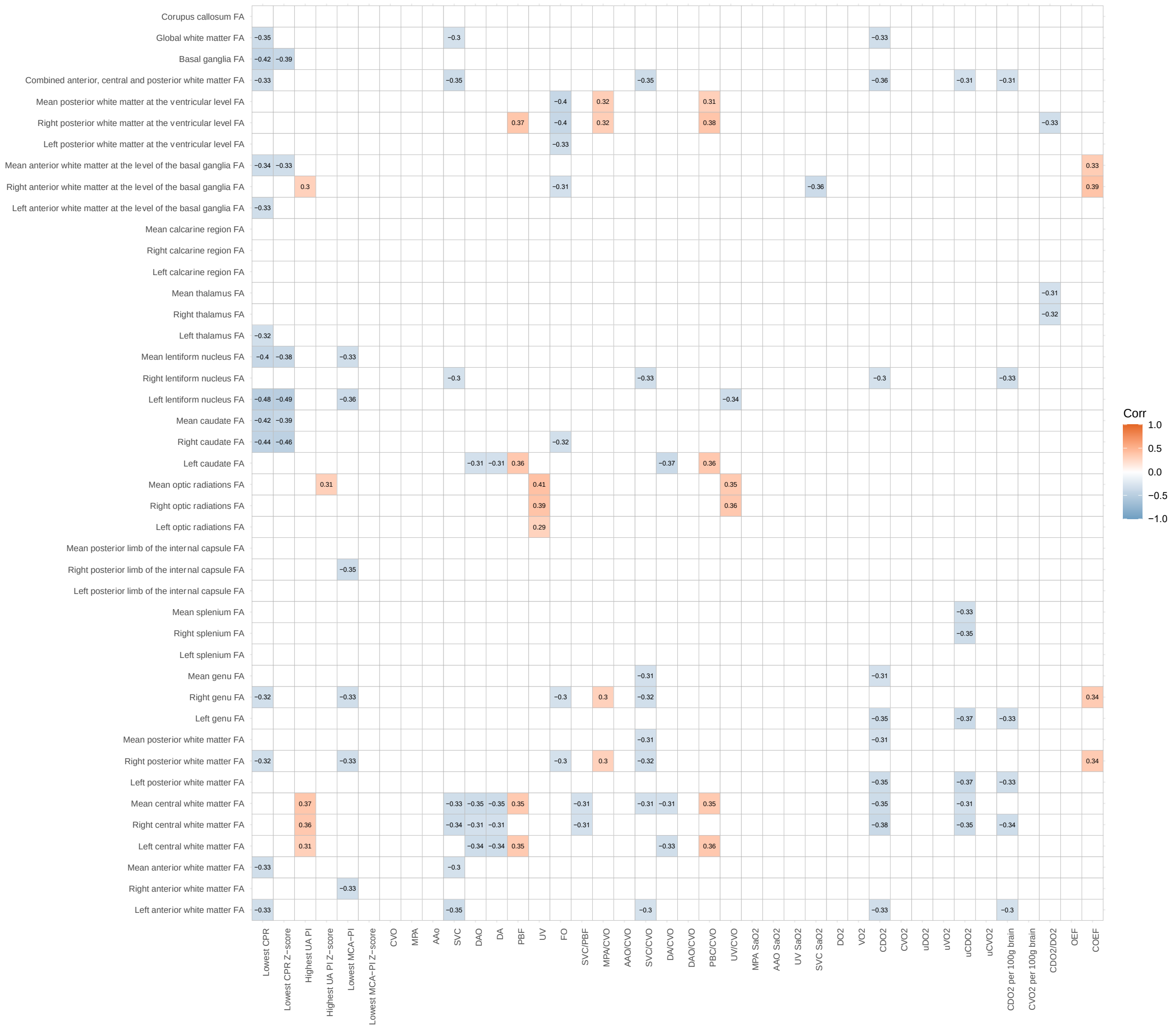

© 2025 Sun L et al. *JAMA Network Open*.

(C)

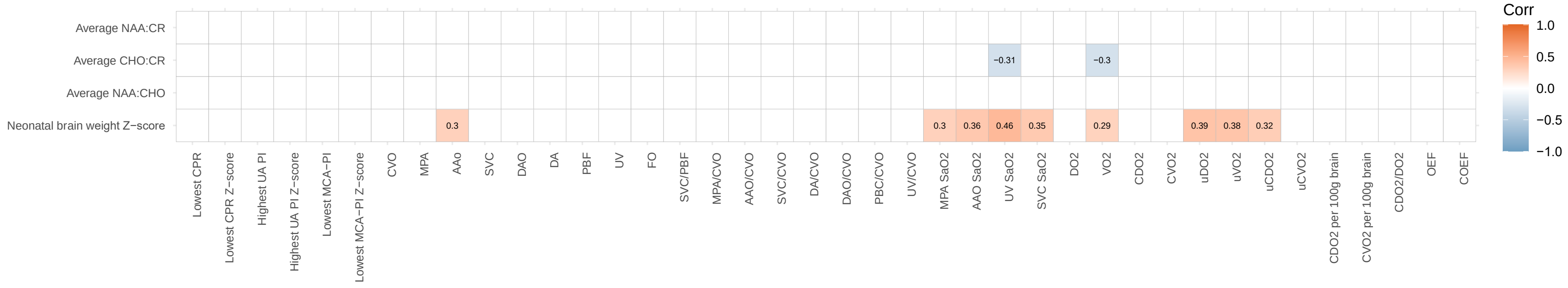

fetal hemodynamics and neonatal diffusion weighted imaging, (B) fetal hemodynamics and neonatal diffusion tensor imaging, (C) fetal hemodynamics with brain weight and magnetic resonance imaging. Significant correlations are shown in colour. ADC, apparent diffusion coefficient; FA, fractional anisotropy; NAA, N-acetylaspartate; CHO, choline; CR, creatine; COEF, cerebral oxygen extraction fraction; OEF, oxygen extraction fraction; CDO2, cerebral oxygen delivery; DO2, fetal oxygen delivery; u, unindexed; CVO2, cerebral oxygen consumption; CDO2, cerebral oxygen delivery; VO2, fetal oxygen consumption; DO2, fetal oxygen delivery; SaO2, oxygen saturation; SVC, superior vena cava; UV, umbilical vein; AAO, ascending aorta; MPA, main pulmonary artery; UV, umbilical vein; PBF, pulmonary blood flow; CVO, combined ventricular output; DAo, descending aorta; DA, ductus arteriosus; FO, foramen ovale; MCA-PI, middle cerebral artery pulsatility index; UA PI, umbilical artery pulsatility index; CPR, cerebroplacental ratio.

© 2025 Sun L et al. *JAMA Network Open*.

eFigure 3. Correlations Between Fetal Hemodynamics and Somatic Growth

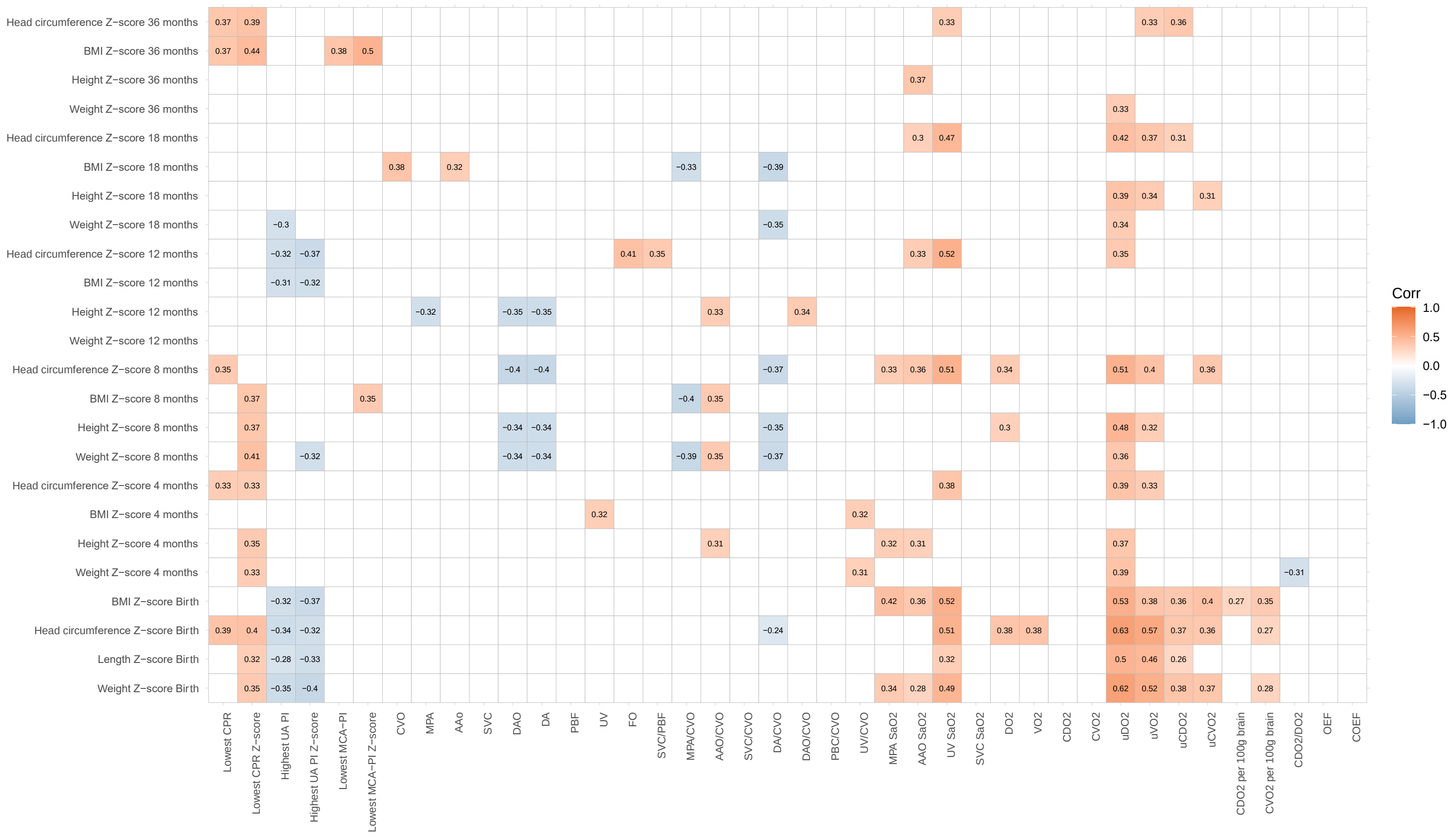

Significant correlations are shown in colour. BMI, body mass index; COEF, cerebral oxygen extraction fraction; OEF, oxygen extraction fraction; CDO2, cerebral oxygen delivery; DO2, fetal oxygen delivery; u, unindexed; CVO2, cerebral oxygen consumption; CDO2, cerebral oxygen delivery; VO2, fetal oxygen consumption; DO2, fetal oxygen delivery; SaO2, oxygen saturation; SVC, superior vena cava; UV, umbilical vein; AAO, ascending aorta; MPA, main

© 2025 Sun L et al. *JAMA Network Open*.

pulmonary artery; UV, umbilical vein; PBF, pulmonary blood flow; CVO, combined ventricular output; DAo, descending aorta; DA, ductus arteriosus; FO, foramen ovale; MCA-PI, middle cerebral artery pulsatility index; UA PI, umbilical artery pulsatility index; CPR, cerebroplacental ratio.

© 2025 Sun L et al. *JAMA Network Open*.

eFigure 4. Correlations Between Fetal Hemodynamics and Neurodevelopmental Outcomes

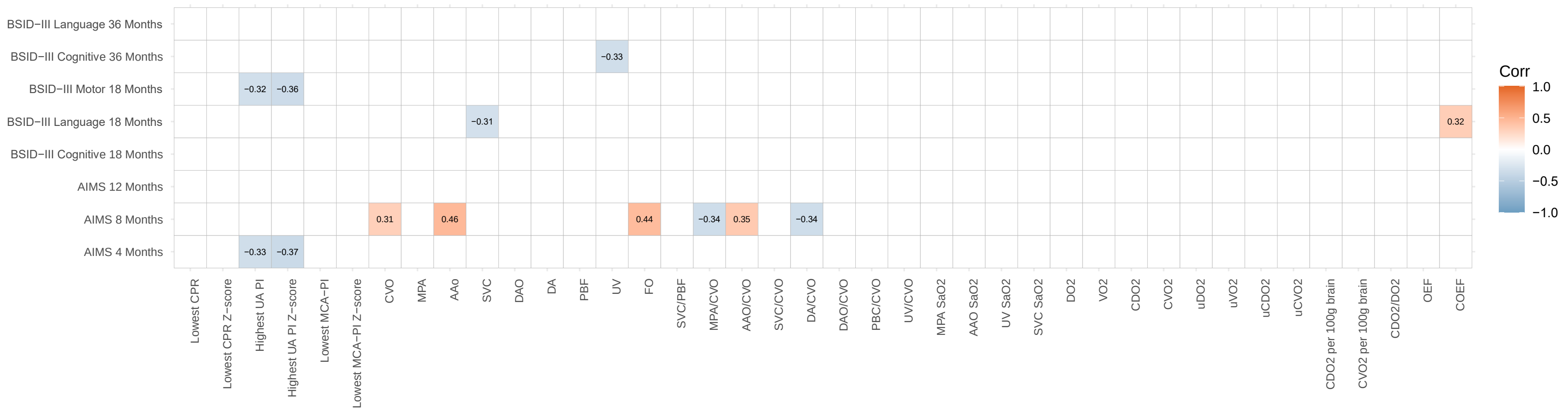

Significant correlations are shown in colour. AIMS, Alberta Infant Motor Scale; BSID-III, Bayley Scales of Infant and Toddler Development 3rd Edition; COEF, cerebral oxygen extraction fraction; OEF, oxygen extraction fraction; CDO2, cerebral oxygen delivery; DO2, fetal oxygen delivery; u, unindexed; CVO2, cerebral oxygen consumption; CDO2, cerebral oxygen delivery; VO2, fetal oxygen consumption; DO2, fetal oxygen delivery; SaO2, oxygen saturation; SVC, superior vena cava; UV, umbilical vein; AAO, ascending aorta; MPA, main pulmonary artery; UV, umbilical vein; PBF, pulmonary blood flow; CVO, combined ventricular output; DAo, descending aorta; DA, ductus arteriosus; FO, foramen ovale; MCA-PI, middle cerebral artery pulsatility index; UA PI, umbilical artery pulsatility index; CPR, cerebroplacental ratio.

© 2025 Sun L et al. *JAMA Network Open*.

eFigure 5. Correlations Between perinatal brain growth and Neurodevelopmental Outcomes

(A)

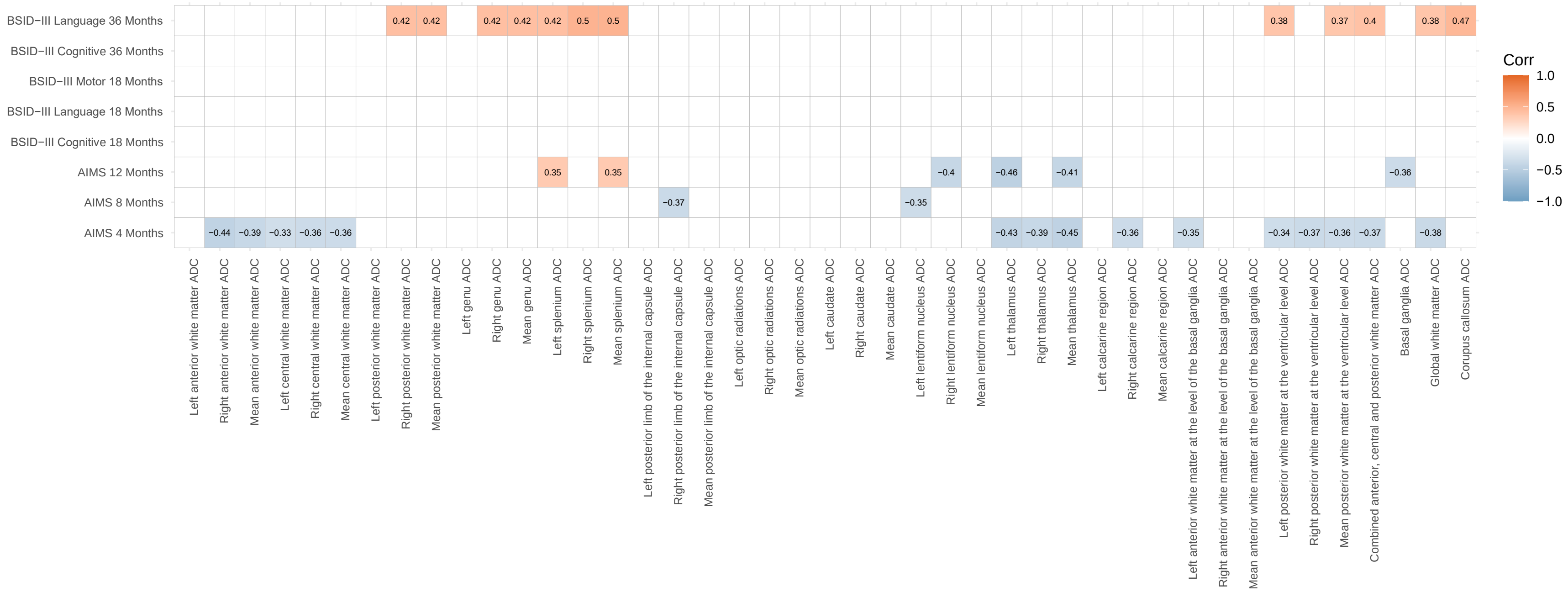

© 2025 Sun L et al. *JAMA Network Open*.

(B)

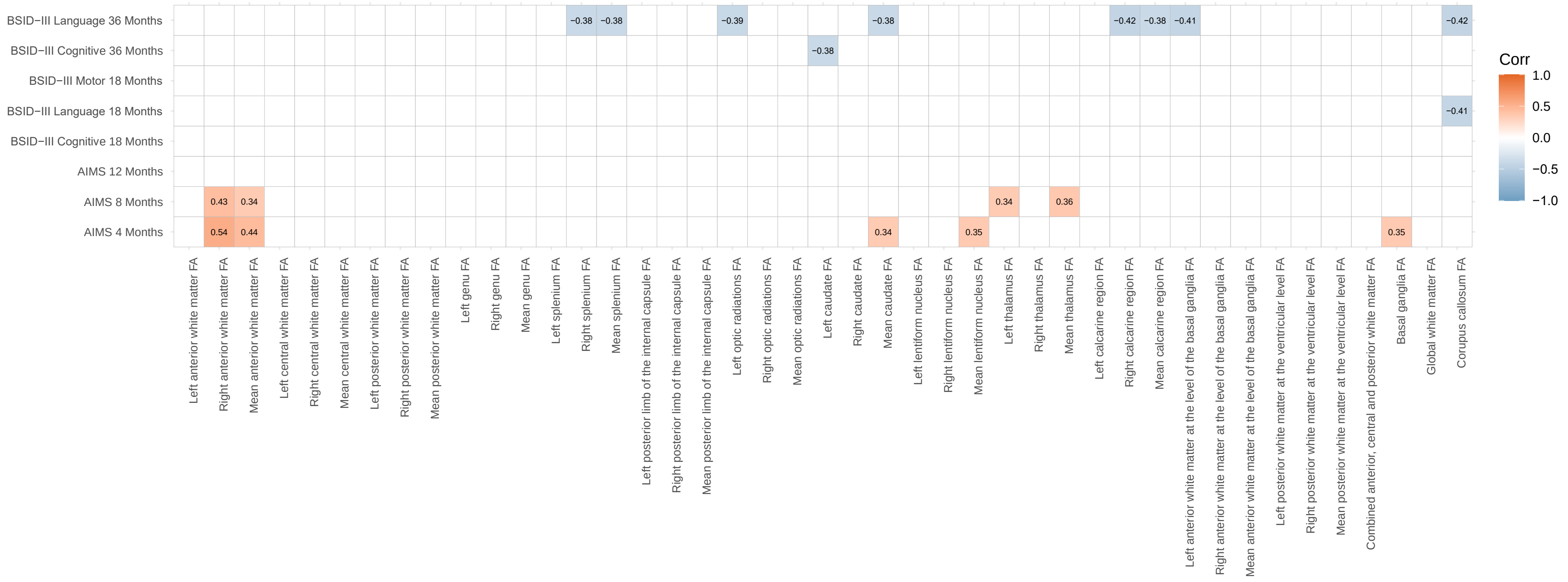

© 2025 Sun L et al. *JAMA Network Open*.

(C)

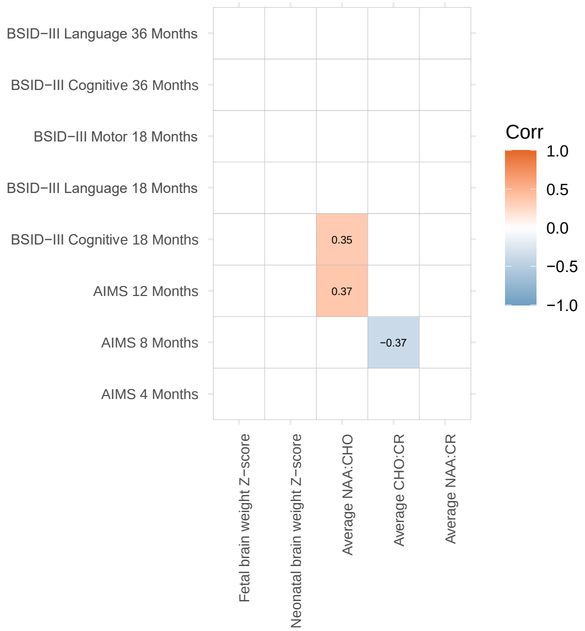

(A) neonatal diffusion weighted imaging and neurodevelopmental outcomes, (B) neonatal diffusion tensor imaging and neurodevelopmental outcomes, (C) brain weight and magnetic resonance imaging with neurodevelopmental outcomes.

Significant correlations are shown in colour. AIMS, Alberta Infant Motor Scale; BSID-III, Bayley Scales of Infant and Toddler Development 3rd Edition; ADC, apparent diffusion coefficient; FA, fractional anisotropy; COEF, cerebral oxygen extraction fraction; OEF, oxygen extraction fraction; CDO2, cerebral oxygen delivery; DO2, fetal oxygen delivery; u, unindexed; CVO2, cerebral oxygen consumption; CDO2, cerebral oxygen delivery; VO2, fetal oxygen consumption; DO2, fetal oxygen delivery; SaO2, oxygen saturation; SVC, superior vena cava; UV, umbilical vein; AAO, ascending aorta; MPA, main pulmonary artery; UV, umbilical vein; PBF, pulmonary blood flow; CVO, combined ventricular output; DAo, descending aorta; DA, ductus arteriosus; FO, foramen ovale; MCA-PI, middle cerebral artery pulsatility index; UA PI, umbilical artery pulsatility index; CPR, cerebroplacental ratio; N-acetylaspartate; CHO, choline; CR, creatine

© 2025 Sun L et al. *JAMA Network Open*.

eFigure 6. Correlations Between Somatic Growth and Neurodevelopmental Outcomes

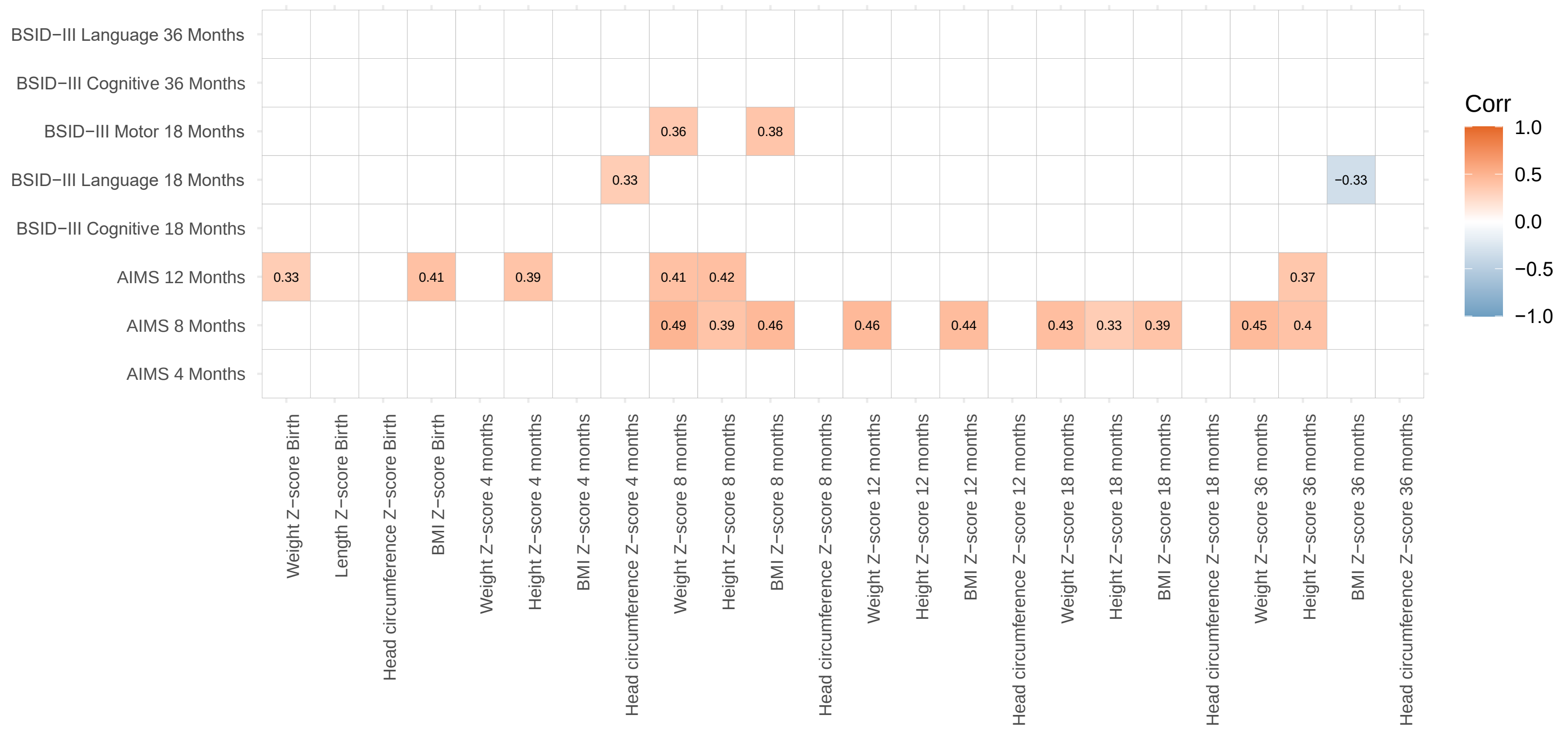

Significant correlations are shown in colour. AIMS, Alberta Infant Motor Scale; BSID-III, Bayley Scales of Infant and Toddler Development 3rd Edition; BMI, body mass index

© 2025 Sun L et al. *JAMA Network Open*.

eReferences.

1. Sun L, Macgowan CK, Sled JG, et al. Reduced Fetal Cerebral Oxygen Consumption Is Associated With Smaller Brain Size in Fetuses With Congenital Heart Disease. *Circulation*. 2015;131(15):1313-1323. doi:10.1161/CIRCULATIONAHA.114.013051

2. Baker PN, Johnson IR, Gowland PA, et al. Fetal weight estimation by echo-planar magnetic resonance imaging. *The Lancet*. 1994;343(8898):644-645. doi:10.1016/S0140-6736(94)92638-7

3. Fenton TR, Kim JH. A systematic review and meta-analysis to revise the Fenton growth chart for preterm infants. *BMC Pediatr*. 2013;13(1):59. doi:10.1186/1471-2431-13-59

4. Roelfsema NM, Hop WCJ, Boito SME, Wladimiroff JW. Three-dimensional sonographic measurement of normal fetal brain volume during the second half of pregnancy. *American Journal of Obstetrics and Gynecology*. 2004;190(1):275-280. doi:10.1016/S0002-9378(03)00911-6

5. Archie JG, Collins JS, Lebel RR. Quantitative Standards for Fetal and Neonatal Autopsy. *Am J Clin Pathol*. 2006;126(2):256-265. doi:10.1309/FK9D5WBA1UEPT5BB

6. Rudolph AM. *Congenital Diseases of the Heart: Clinical-Physiological Considerations*. 3rd ed. Wiley-Blackwell; 2009.

7. Nicolaides KH, Clewell WH, Mibashan RS, Soothill PW, Rodeck CH, Campbell S. FETAL HAEMOGLOBIN MEASUREMENT IN THE ASSESSMENT OF RED CELL ISOIMMUNISATION.

8. Wright GA, Hu BS, Macovski A. Estimating oxygen saturation of blood in vivo with MR imaging at 1.5 T. *Magnetic Resonance Imaging*. 1991;1(3):275-283. doi:10.1002/jmri.1880010303

9. Lim JM, Porayette P, Marini D, et al. Associations Between Age at Arterial Switch Operation, Brain Growth, and Development in Infants With Transposition of the Great Arteries. *Circulation*. 2019;139(24):2728-2738. doi:10.1161/CIRCULATIONAHA.118.037495

10. Brummelte S, Grunau RE, Chau V, et al. Procedural pain and brain development in premature newborns. *Annals of Neurology*. 2012;71(3):385-396. doi:10.1002/ana.22267

11. Chau V, Poskitt KJ, McFadden DE, et al. Effect of chorioamnionitis on brain development and injury in premature newborns. *Annals of Neurology*. 2009;66(2):155-164. doi:10.1002/ana.21713

© 2025 Sun L et al. *JAMA Network Open*.
