# Supplementary material for: Neurodevelopment Among Infants With Late-Onset Fetal Growth Restriction
Source: JAMA Netw Open. 2025 Jun 25;8(6):e2517360. doi: 10.1001/jamanetworkopen.2025.17360 (PMC12199057; doi:10.1001/jamanetworkopen.2025.17360)
Supplement: Supplement 2. — Data Sharing Statement [file jamanetwopen-e2517360-s002.pdf]

Data Sharing Statement

Sun. Neurodevelopment Among Infants With Late-Onset Fetal Growth Restriction. *JAMA Netw Open*. Published June 25, 2025. doi:10.1001/jamanetworkopen.2025.17360

Data
Data available: No

Additional Information
Explanation for why data not available: The data that support the findings of this study are available from the corresponding author upon reasonable request.
